# Integrating Machine Learning With Constant‐Potential Simulation to Unravel Charge‐Transfer Mechanisms in Electrochemical Nitrogen Fixation

**DOI:** 10.1002/advs.202524356

**Published:** 2026-03-13

**Authors:** Yufei Xue, Dushuo Feng, Yuefei Zhang, Yang Zhang, Yalong Jiao, Aijun Du, Guoping Gao

**Affiliations:** ^1^ MOE Key Laboratory for Non‐equilibrium Synthesis and Modulation of Condensed Matter Shaanxi Province Key Laboratory of Advanced Functional Materials and Mesoscopic Physics School of Physics Xi'an Jiaotong University Xi'an Shaanxi China; ^2^ Department of Sport and Exercise Science College of Education Zhejiang University Hangzhou China; ^3^ College of Physics Hebei Key Laboratory of Photophysics Research and Application Hebei Normal University Shijiazhuang China; ^4^ School of Chemistry and Physics and Centre for Material Science Faculty of Science Gardens Point Campus Queensland University of Technology Brisbane Queensland Australia

**Keywords:** density functional theory, electrochemical nitrogen reduction reaction, machine learning, single‐atom catalysts

## Abstract

The electrochemical nitrogen reduction reaction (NRR) offers a sustainable approach to ammonia (NH_3_) synthesis under mild conditions. To achieve scalable NH_3_ production, discovering high‐performance catalysts for the efficient NRR is crucial. For this purpose, the activity mechanisms of functional group‐modified carborin/graphene‐supported single‐atom catalysts were systematically investigated using the grand‐canonical fixed‐potential method, which simulates operando constant‐potential conditions. Among 144 candidates screened, Cr@NO_2_‐carborin/graphene and Cr@CHO‐carborin/graphene are identified as the most promising NRR catalysts, with low limiting potentials of −0.220 V for the ^*^N_2_→^*^N_2_H step and −0.245 V for the ^*^NH→^*^NH_2_ step, respectively. Furthermore, interpretable machine learning models revealed that the shift in potential of zero charge, induced by intermediate adsorption, serves as the key voltage‐responsive descriptor governing charge transfer patterns and influencing the N_2_ activation. These findings establish a paradigm shift from static electronic descriptors to dynamic interfacial property engineering, offering a universal framework for designing electrocatalysts for multi‐electron reactions like NRR.

## Graphical abstract

Integrating interpretable machine learning with the fixed‐potential method reveals a novel mechanism: the catalytic activity of the electrochemical nitrogen reduction reaction is governed by partial charge transfer, induced by variations in the intermediate potential of zero charge under constant potential.

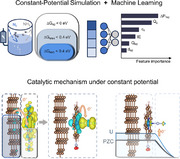

## 1 Introduction

Ammonia synthesis via nitrogen reduction reaction (NRR) is pivotal for sustainable energy systems [1, 2]. However, current industrial methods rely on the century‐old Haber‐Bosch process for ammonia production, which heavily depends on fossil fuels under high temperatures and pressures [3, 4], accounting for nearly 2% of global energy consumption and 3% of global CO_2_ emissions [5]. As an alternative strategy, electrochemical nitrogen reduction using catalysts is a promising approach for sustainable, low‐energy, zero‐CO_2_ emission, distributed ammonia production. Moreover, the development of efficient electrocatalysts for NRR faces formidable challenges. Due to the sluggish kinetics of N_2_ dissociation caused by the high bond energy of the N≡N triple bond (941 kJ/mol) and the severe competition from the hydrogen evolution reaction (HER) in aqueous electrolytes, the NRR exhibits low activity and selectivity [6, 7, 8, 9].

To address these challenges, the single‐atom molecular catalysts (SAMCs) with isolated metal atoms firmly anchored in non‐metallic frameworks (e.g., organic ligands or molecular structures), emerge as a pivotal research area in various catalytic processes [10]. These systems exhibit significant promise for enhancing NRR selectivity due to their atomic‐level precision. Specifically, SAMCs facilitate atomic‐level modulation of the coordination environment through functional group modifications or ligand design, while simultaneously enabling multifunctional catalysis. Recent advancements in this area include work conducted by Xia Hai's group, who has synthesized a series of single‐atom molecules based on the carbolong family. These molecules consist of a conjugated π‐framework, which features a unique class of carbon‐based molecular skeletons composed of pure carbon chains with 7–15 carbon atoms integrated with metal centers [11, 12, 13, 14]. The carbolong family has provided a versatile platform for synergistic catalysis. For example, bimetallic carbolong complexes enable alkene difunctionalization to mediate the dehydrogenation of alcohols [15]. Carborin, a newly synthesized carbolong molecule with a transition metal centered among up to five aromatic rings in D_5_h symmetry and containing 15 carbon atoms [16], offers a promising and multifaceted platform for NRR research.

To screen and optimize the NRR performance of SAMCs through density functional theory (DFT) simulations, a reliable evaluation of catalytic activity should consider not only the solution effects (i.e., solvation effects) but also two other critical factors: the voltage effect and the SAMC‐substrate interaction. First, under realistic working conditions, voltage‐driven charge redistribution at electrified interfaces induces noticeable changes in the free energy profile of intermediate systems [17, 18, 19]. These altered free‐energy profiles are crucial to the six‐electron NRR pathway, which involves sequential proton‐coupled electron transfers (PCET) through multiple metastable intermediates (e.g., ^*^N_2_H, ^*^NNH_2_, ^*^N, ^*^NH_2_; asterisk denotes adsorption site). Second, SAMCs typically exhibit low intrinsic conductivity [20]. When interfaced with conductive substrates (e.g., graphene, carbon nanotubes), hybrid interfaces display non‐negligible electronic coupling effects, including structural distortions, metal center charge state changes, and substrate‐dependent interfacial polarization. These effects are expected to reshape the reaction pathways for NRR. High‐throughput computational approaches integrating interpretable machine learning (ML) with the fixed‐potential method (FPM) can not only predict the most promising NRR catalysts but also probe the potential effects during NRR.

Herein, our DFT data for NRR are obtained based on a heterojunction composed of a graphene substrate and TM@FG‐carborin molecules (TM and FG are the transition‐metal and the functional group, respectively). To enlarge the sample size and adjust the activation energy of the intermediate reactions, H atoms on the aromatic rings are substituted with different functional groups. Through systematic screening, Cr@NO_2_‐carborin/graphene and Cr@CHO‐carborin/graphene are identified as the most promising NRR catalysts. By integrating FPM with ML, we not only map the relationships between diverse chemical/physical characteristics and NRR catalytic performance, but also uncover a groundbreaking mechanism: catalytic activity is governed by partial charge transfer, which is induced by variations in the intermediate potential of zero charge (PZC) under constant potential. Our work can provide experimental groups with a clearer picture of the potential effects in the NRR and offers ML‐driven insights to guide high‐performance catalyst design.

## 2 Computational Method

### 2.1 Details of First‐Principles Calculations

A TM@FG‐carborin molecule adsorbed on the $6\times 6\sqrt{3}\times 1$ rectangular supercell of graphene was used as a model to evaluate the NRR performance. To mimic the solvent effects and alleviate Coulomb interactions originating from periodic replicas in an electrochemical environment under applied potentials, we implemented the implicit solvent model with dielectric continuum approximation (with a water dielectric constant ε = 78) in vacuum regions [21]. All the DFT calculations, including structural optimization and ab initio molecular dynamics (AIMD), were conducted using the PWmat package incorporating the grand canonical FPM [22, 23]. The AIMD simulations were conducted at 300 K for 5,000 fs with a 1 fs timestep using Nosé–Hoover thermostats in the canonical ensemble [24, 25]. Atomic charge transfer was analyzed via the Hirshfeld method [26, 27, 28, 29, 30]. More computational details can be found in Note S1 of the Supporting Information.

Even with similar equilibrium potentials, NRR requires a significantly higher electron consumption of 6 *e^−^* to reduce N_2_ to 2NH_3_ compared to HER, which consumes only 2 *e^−^* per H_2_ molecule produced. This inherent disparity in electron demand underscores the critical need for precise modeling of potential‐dependent charge transfer in multi‐step mechanisms. The widely‐used computational hydrogen electrode (CHE) approach is based on an ideal assumption that one electron transfers from the reservoir to the charge‐neutral system per elementary step [31, 32]. In contrast, FPM is capable of precisely calculating different charged states of reaction intermediates based on the target potential. Details of the method can be found in the Reference: Journal of Catalysis 391 (2020) 530–538 [19].

We therefore use the first PCET step of NRR to illustrate the difference in Gibbs free energy calculation between the FPM and the CHE approach. Within the CHE, this step can be represented as:

(1) $$*N_{2}+\left(H^{+}+e^{-}\right)=*\mathrm{NNH}$$

In contrast, the FPM accounts for the varying PZC induced by the adsorption of various intermediates (i.e., ^*^N_2_, ^*^NNH, ^*^N, ^*^NH_3_ etc.) onto catalysts. These PZC variations lead to different charge states of the system at a constant applied potential. If the total charge is *n*_1_ before the reaction and *n*_2_ after the reaction, the same PCET step within FPM is described as

(2) $$*N_{2}^{(n_{1}+)}+H^{+}+\left(1+n_{1}-n_{2}\right)e^{-}=*{\mathrm{NNH}}^{(n_{2}+)}$$

This formulation reveals a critical distinction: the net charge transfer in the FPM is (*n*_2_−*n*_1_−1)*e*, compared to a fixed charge transfer of –1e in CHE model Equation (1). This difference in treating charge transfer results in a different Gibbs free energy calculation formula. In the FPM, the Gibbs free energy for this step can be computed by

(3) $$\Delta G=\Delta E_{\mathrm{tot}}-T\Delta S+\Delta E_{\mathrm{ZPE}}+\left(n_{2}-n_{1}-1\right)eU_{\mathrm{abs}}$$

where △*E_tot_* represents the total energy difference between two reaction steps. *T*△*S* and △E_ZPE_ are the variations in entropy and zero‐point energy (ZPE), respectively. The gas entropies *S* of N_2_, NH_3,_ and H_2_ are obtained at room temperature (*T* = 298.15 K) and atmospheric pressure [33]. The final term explicitly accounts for the electrical energy consumption induced by charge transfer in the elementary step under the applied potential (*U_abs_*). The CHE framework simplifies this to e ∙ *U_abs_*. Although this simplification is effective for reactions with straightforward mechanisms, it becomes inadequate for modeling complex electrochemical interfaces, where accurately describing the evolving charge state at each step is crucial. Neglecting this complexity can even lead to errors in identifying the rate‐determining step [34, 35]. Consequently, the integration of ML with FPM establishes a transformation framework for systematically evaluating how multiple chemical and physical factors (i.e., PZC, electronegativity, and structural parameters) contribute to reaction performance under constant‐potential operational conditions.

### 2.2 Details of Machine Learning Models

The efficient and reliable nonlinear ML models are essential for uncovering potential effects in catalysis. To ensure model robustness and generalization, the dataset was partitioned into an 80% training set and a 20% test set. The performance of the ML algorithms was quantitatively assessed using two metrics: the Root Mean Square Error (RMSE) and the Coefficient of Determination (R^2^ score), defined as follows:

(4) $$\mathrm{RMSE}=\sqrt{\frac{1}{n}\sum_{i}^{n}{\left(f\left(x_{i}\right)-y_{i}\right)}^{2}}$$

(5) $$R^{2}=1-\frac{\sum_{i}^{n}{\left(y_{i}-f\left(x_{i}\right)\right)}^{2}}{\sum_{i}^{n}{\left(y_{i}-\bar{y}\right)}^{2}}$$

where *n* is the number of data points, *f*(*x_i_*) represents the predicted value, *y_i_* is the DFT value;$\bar{y}$ is the mean of DFT values. The testing results of RMSE and R^2^ score for all regression models used to predict adsorption energies *∆G_N2_* and *∆G_NNH_* are shown in Note S2 and Figures S2 and S3. In the main text, the Least Squares Boosting Regression (LSBoost) model was chosen for explainable ML analysis due to its robustness and interpretability (RMSE = 0.051, R^2^ = 0.995 for *∆G_N2_* prediction, and RMSE = 0.321, R^2^ = 0.878 for *∆G_NNH_* prediction) [36, 37]. Moreover, the Pearson correlation analysis was employed to quantify the linear dependence between ML features using the following formula:

(6) $$p=\frac{\sum_{i}\left(x_{i}-\bar{x}\right)\left(y_{i}-\bar{y}\right)}{\sqrt{\sum_{i}{\left(x_{i}-\bar{x}\right)}^{2}}\sqrt{\sum_{i}{\left(y_{i}-\bar{y}\right)}^{2}}}$$

where *x* and *y* are two feature values, $\bar{x}$ and $\bar{y}$ denote their corresponding means. In addition, SHapley Additive exPlanations (SHAP) analysis was applied to interpret the model's predictive behavior [38, 39, 40]. The sign of each SHAP value (positive/negative) indicates whether a feature enhances or suppresses the predicted outcome, and the absolute magnitude of the SHAP value reflects its relative contribution on model output. The significance of this ML framework primarily lies in its comprehensive capabilities. Not only does it enable the prediction of catalysts with high NRR activity, but it also facilitates the elucidation of the underlying catalytic reaction mechanisms.

## 3 Results and Discussion

**FIGURE 1:**
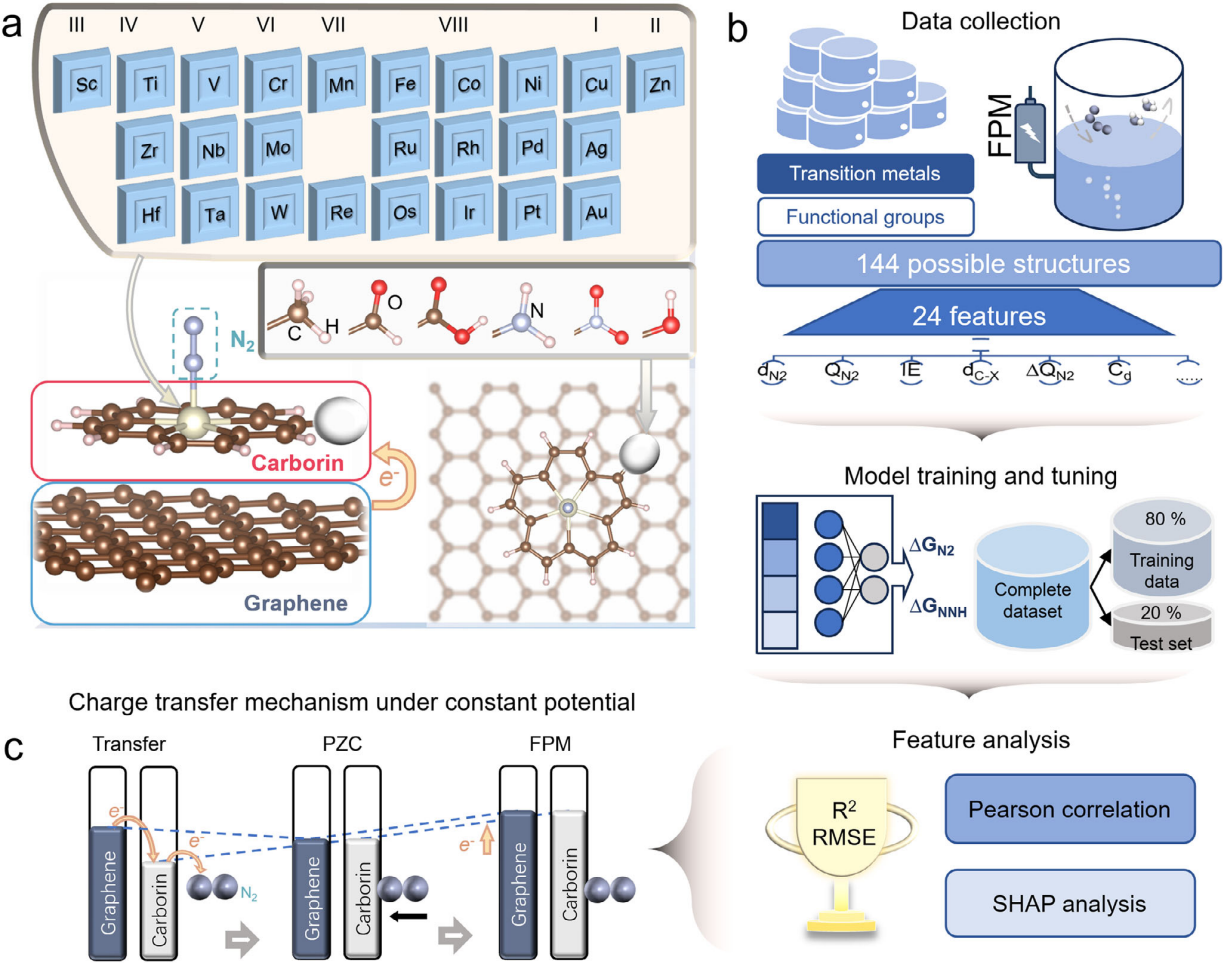
(a) Side and top views of TM@FG‐carborin/graphene (TM = transition metal, FG = functional group), where carborin is a metal‐centered planar annulene. (b) Workflow of the DFT‐ML screening for NRR. The process includes data collection, model training and tuning, feature analysis and prediction, and explanation of the charge transfer mechanism. (c) Diagram of PZC and applied potential via FPM in determining the charge transfer dynamics and its impact on N_2_ activation.

Based on the computational methods described above, we constructed a heterojunction framework consisting of a graphene substrate and TM@FG‐carborin molecules (TM = transition metal, FG = functional group). We selected most transition metals, among which Tc was excluded due to its radioactivity, yielding a total of 25 metals. During the subsequent structural relaxation, we carefully inspected each optimized structure individually and found that a stable 5‐coordinate C structure could hardly be formed with Cu as the central metal atom (see Figure S4); thus, these configurations were discarded. Combining these structural motifs with the six functional groups (─CH_3_, ─CHO, ─COOH, ─NH_2_, ─NO_2,_ and OH), 144 candidate catalysts were generated for an interpretable ML study as illustrated in Figure 1a. To probe their catalytic behavior, we simulated the processes of N_2_ adsorption and the first PCET step on the above catalysts via grand canonical FPM under constant potential. This approach gathered catalytic features and descriptors necessary for subsequent ML analysis to elucidate their influence mechanisms on NRR activity. Through systematic feature engineering, we identified 24 features and categorized them into three groups according to their physical and chemical properties for Pearson analysis:

1) the features characterizing charge distribution of optimized intermediates: including the charge states for different intermediates (Q_*_, Q_*N2_, and Q*_NNH_), the charge transfer during N_2_ adsorption (∆Q_N2_ = Q_*N2_ – Q_*_) and the first PCET step (*∆Q_NNH_*), and the Hirshfeld charge changes of graphene (*Q_g_*), five C coordination atoms of TM atom (*Q_5C_*), functional group (*Q_fg_*), TM atom (*Q_x_*), and N_2_ molecule (*Q_N2_*) during N_2_ adsorption. The orbital hybridization is characterized by the band centre of TM‐d orbital and N‐p orbital (*c_d_* and *c_p_*), band centre difference and ratio (*c_d_*‐*c*_p_ and *c_d_*/*c_p_*). These features provide a microscopic insight into potential effects on charge transfer among interlayers, coordination atoms, functional groups, and TM centers.

2) the features characterizing atomic structures: the average distance between TM atom and coordination atoms (*d_C‐X_*), the bond length between functional group and carborin (*d_fg_*), the length of the shorter N‐TM bond in _*_N_2_ (*d_N2_*). These atomic‐structure‐related features collectively offer valuable information about the geometric configuration and interaction environment of the catalyst system, which are fundamental for predicting and analyzing its catalytic performance.

3) The electron transfer capabilities are characterized by: electronegativity (*EN*), the first ionization energy (*IE*) of TM, the PZCs of *, *N_2_, and *NNH systems (P_*_, P_*N__2,_ and P_*NNH_), and PZC changes during N_2_ adsorption (∆P_*N2_) and the first PCET step (∆P_*NNH_). These features directly associate with activation of N_2_ and NNH, providing a comprehensive framework for understanding catalytic mechanisms.

**FIGURE 2:**
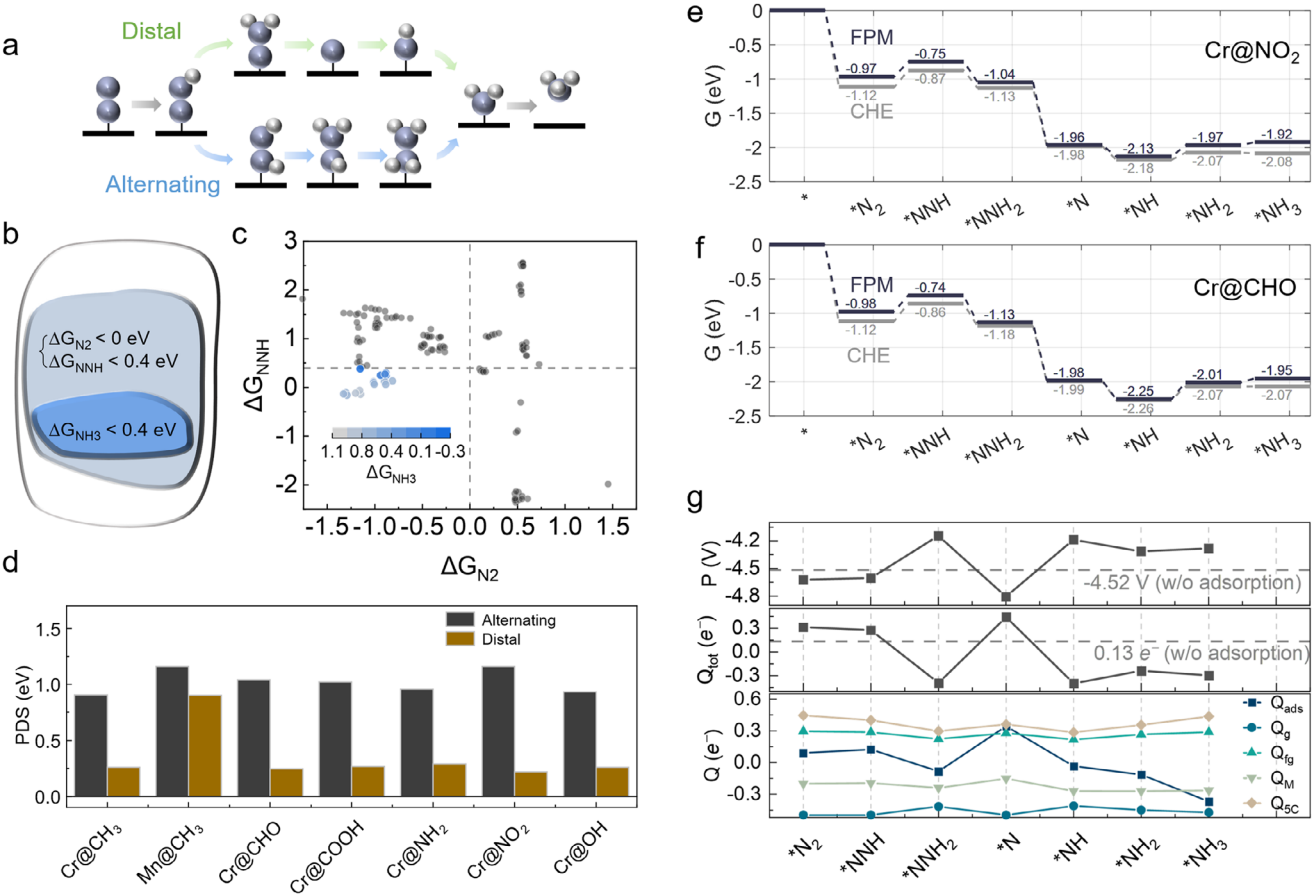
(a) The distal and alternating pathways for NRR. (b) Schematic diagram of three‐step free energy screening for optimal catalysts. (c) The free energy changes for the N_2_ adsorption (*∆G_N2_*) and the first hydrogenation (*∆G_NNH_*), and the color circles in the region where *∆G*_N2_ < 0 eV and *∆G_NNH_* < 0.4 eV, are mapped to the *∆G_NH3_* values. (d) The potential determining steps (PDS) of alternating and distal pathways for seven catalysts. (e‐f) The Gibbs free energy diagrams of NRR pathways for the two best catalysts (Cr@NO_2_‐carborin and Cr@CHO‐carborin) obtained by FPM at *U_abs_* = –4.44 V and CHE. (g) Electronic charge variation for Cr@NO_2_‐carborin, where positive and negative values represent the electrons gained and lost, respectively. The dashed lines indicate the catalyst parameters as a reference.

As illustrated in Figure 2a, the adsorption of N_2_ and the first PCET step (^*^N_2_ → ^*^NNH) are essential steps for both the distal and alternating pathways of NRR [41]. According to the Sabatier principle, the NRR activity is significantly constrained by the linear scaling relationship between the energy profiles of two key intermediates ^*^N_2_H and ^*^NH_2_ [42]. Thus, we set about fast‐screening NRR candidates with the three primary criteria: 1) the N_2_ adsorption energy (* → *N_2_) *∆G_N2_* < 0 eV; 2) the reaction energy of the first PCET step (*N_2_ → *NNH) *∆G_NNH_* < 0.4 eV; and 3) the reaction energy of the final PCET step (^*^NH_2_ → ^*^NH_3_) *∆G_NH3_* < 0.4 eV, as shown in Figure 2b. The first criteria ensures spontaneous N_2_ adsorption (a prerequisite for NRR initiation), while the second and third ones are set to avoid excessively high energy barriers for key intermediate formation and product desorption based on the NRR fundamental reaction mechanism and relevant literature precedents [43]. These three criteria allow us to screen NRR catalysts rapidly. In addition, we note that the experimental operating potential for NRR is affected by the specific experimental environment, and the discussion on the importance of descriptors in this manuscript is not affected by the choice of this potential based on our preliminary test. Therefore, we employed the commonly used standard hydrogen electrode potential of *U_abs_* = −4.44 V as a reference. Based on the first and second criteria, we screened out 19 catalysts as shown in Figure 2c. Furthermore, we applied a more stringent filtering of the third criterion to yield 7 optimal catalysts: graphene‐supported Mn@CH_3_‐carborin, Cr@CH_3_‐carborin, Cr@CHO‐carborin, Cr@COOH‐carborin, Cr@NH_2_‐carborin, Cr@NO_2_‐carborin, and Cr@OH‐carborin, as highlighted by the blue circles in Figure 2c. To clarify the origin of Cr's superior NRR performance, we compared the ^*^NNH adsorption energy and metal *d*‐band center of 8 transition metals (Sc, Ti, V, Cr, Mn, Fe, Co, Ni) under identical carborin ligand, graphene support, and functional group conditions, see Figure S6. There is a distinct inverted‐V (volcano‐type) trend in ^*^NNH adsorption energy with respect to the metal *d*‐band center. Cr is precisely at the curve bottom, showing the most favorable ^*^NNH binding strength—balancing intermediate activation and product desorption. This consistent trend confirms Cr's advantage stems from its intrinsic optimal *d*‐band electronic structure, not functional group type.

To further investigate the electrochemical catalytic performance of these seven catalysts, we computed the potential‐determining step (PDS) values for both the distal and alternating NRR reaction pathways as illustrated in Figure 2d. The ^*^N_2_H intermediate undergoes further hydrogenation to form two distinct ^*^N_2_H_2_ species: ^*^NNH_2_ (via the distal pathway) and ^*^NHNH (via the alternating pathway). The distal pathway is energetically favorable for NRR, while the alternating route requires significantly higher PDS for the formation of ^*^NHNH. This disparity in PDS indicates that the distal pathway is kinetically more favorable for catalytic nitrogen reduction. The free energy profiles of the two catalysts with the lowest PDS energies, Cr@NO_2_‐carborin and Cr@CHO‐carborin, are present in Figure 2e,f, respectively. For Cr@NO_2_‐carborin, the PDS occurs at the N_2_→NNH step with a PDS of only 0.220 eV under *U_ab_*_s_ = –4.44 V (the absolute potential of the ideal standard hydrogen electrode, where the potential of a place far away from the electrode is set to zero). In contrast, for Cr@CHO‐carborin, the NRR activity is determined by the penultimate PCET step (^*^NH→^*^NH_2_) with a PDS of 0.245 eV. For a comprehensive evaluation, the free energy diagrams of the remaining five catalysts are systematically compared in Figure S7. In contrast to these catalysts, though some conventional bulk metal catalysts has low PDS for NRR, the inefficiency of NRR is generally attributed to poor selectivity due to dominant HER [44]. Notably, according to Boltzmann distribution analysis, these two catalysts (Cr@NO_2_‐carborin and Cr@CHO‐carborin) achieve up to 99% Faradaic efficiency for NRR over HER, outperforming conventional transition metal catalysts (see details in Note S3). Furthermore, the excellent thermodynamic stability of these two catalysts was confirmed through AIMD simulations conducted at 300 K for 5000 fs, as depicted in Figure S8. In addition, the reaction energy of N_2_ + 3H_2_ = 2NH_3_ was calculated to be –0.83 eV, consistent with previous theoretical results. We also computed the reaction energy for N_2_ + 3H_2_ + H_2_O = 2NH_2_·H_2_O to be –1.58 eV. This value is only ∼0.4 eV higher than the free energy of the *NH_3_ intermediate, indicating that the desorption of NH_3_ from the catalyst surface is thermodynamically achievable.

To verify the influence of potential effect on NRR, we compared the free‐energy results calculated by the conventional CHE (the gray line) and FPM (the black line) as shown in Figure 2e,f. The discrepancies in intermediates' free energy values visually confirm this effect. First, the N_2_ binding strength with the catalyst obtained by FPM is weaker than that of CHE. This is attributed to a shift in the PZCs, which decreases from –4.52 to –4.62 V upon N_2_ adsorption for Cr@NO_2_‐carborin (Figure 2g). This decrease in PZC induces the accumulation of a negative charges state (*Q_tot_*) from 0.132 *e^–^* to 0.316 *e^–^* under the constant absolute potential *U_abs_* = −4.44 V, resulting in weakened N_2_ adsorption. Additionally, the electronic charge variation for Cr@CHO‐carborin, as shown in Figure S9, further supports this conclusion. Second, for the first PCET step (^*^N_2_ → ^*^NNH), the reaction energy obtained by FPM (∼0.220 eV) is slightly smaller than that from CHE (∼0.242 eV). That difference arises because, within FPM calculation the *Q_tot_* of ^*^N_2_ (0.316 *e^–^*) is slightly larger than that of ^*^NNH (0.276 *e^–^*), resulting in a larger number of electrons (*n*_2_ – *n*_1_ – 1) transferred than in CHE (–1 *e^–^*) as described by Equations (1) and (2) and a reduction in the reaction energy. Third, neglecting the actual charged states of intermediates not only leads to inaccurate free energy but can also result in misidentifying the PDS. For instance, in the Cr@CHO‐carborin case, as shown in Figure 2f, the FPM method identified the ^*^NH to ^*^NH_2_ reaction step as the PDS, whereas the CHE method incorrectly points to the PCET process from ^*^N_2_ to ^*^NNH.

**FIGURE 3:**
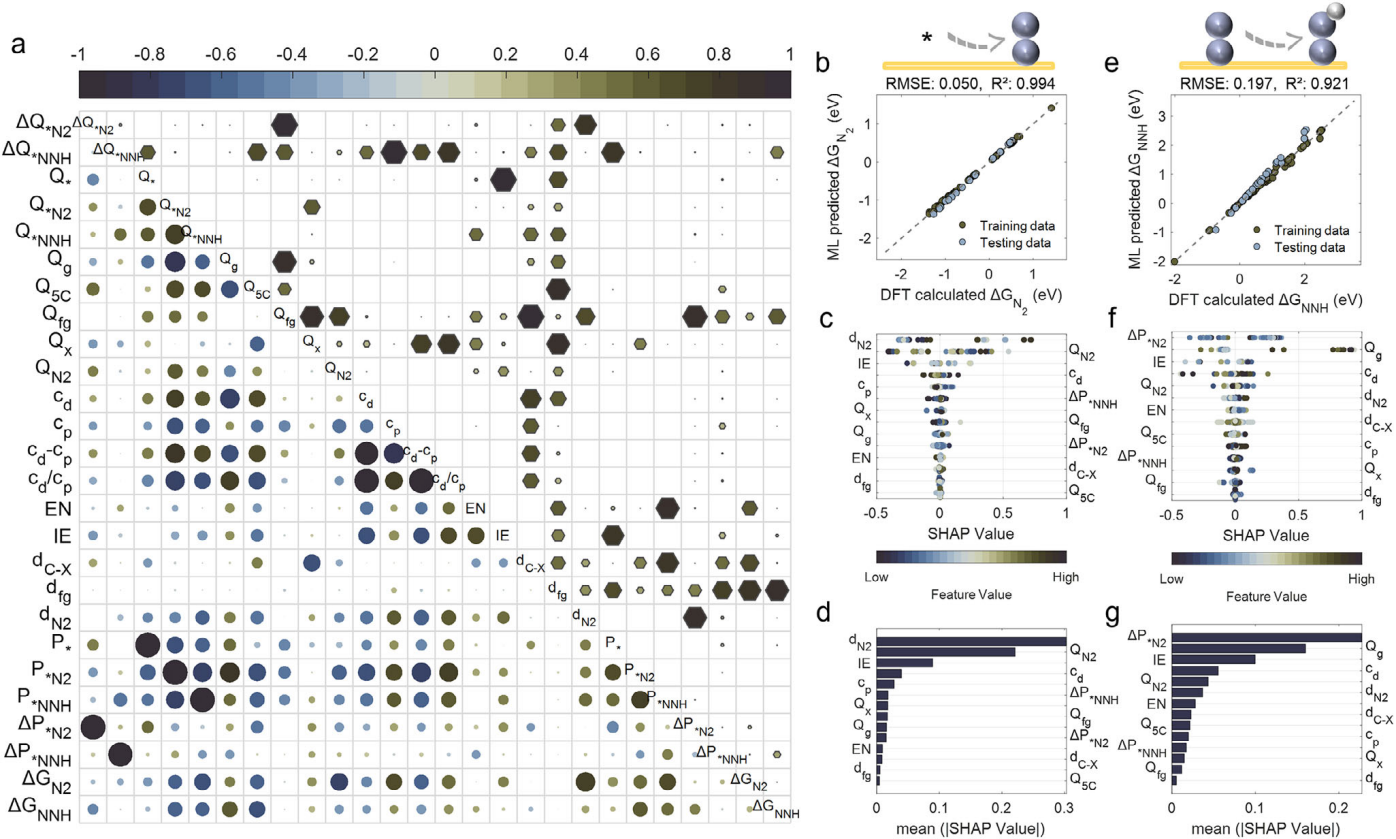
Pearson and SHAP analysis for evaluating the importance of all features under constant potential *U_abs_* = −4.44 V. (a) Pearson correlation plots of 24 features, where the lower‐triangular region displays the correlation coefficients and the upper‐triangular region indicates the significance levels. (b) DFT calculated adsorption energy of N_2_ (∆G_N2_) versus ML predicted values. (c) The distribution of SHAP values and (d) mean SHAP values for each feature in predicting ∆G_N2_. (e)‐(g) Prediction performance and SHAP analysis for reaction free energy difference between ^*^N_2_ and ^*^NNH.

To identify key predictive factors, including PZCs, charge transfer dynamics, and other 12 potential features, for NRR activity, we integrated constant‐potential DFT data with ML. The correlations between these features for the ^*^N_2_ system are quantified by Pearson coefficients and visualized in the heatmap of Figure 3a. This heatmap is structured as follows: the lower‐triangular region uses circles, scaled and colored by the magnitude of the correlation coefficient, to represent the strength of linear relationships; the upper‐triangular region uses hexagons, scaled by size, to encode the corresponding p‐values, where a smaller symbol denotes greater statistical significance. The correlation coefficients between these features and N_2_ adsorption energy ∆G_N2,_ and the reaction energy ∆G_NNH_ are presented in the final two rows of the heatmap. For ∆G_N2_, the highest correlations are *Q_N2_* and *d_N2_* with the highest absolute values of Pearson correlation coefficients of 0.71 and 0.78, respectively. In contrast, the conventional electronic structure features, d‐band center *c_d_* and the band center difference between TM‐*d* and N‐*p* orbitals *c_d_‐c_p,_* show more moderate correlations under the FPM framework, with Pearson correlation coefficients of −0.42 and −0.58, respectively. For ∆G_NNH_, the most significant correlations are with the charge change on the graphene substrate (Q_g_, with a Pearson correlation coefficient of 0.60) and the charge change on the coordination atoms (*Q_5C_*, with a Pearson correlation coefficient of –0.66), while the Pearson correlation coefficient of *c_d_‐c_p_* and c_d_ are –0.36 and 0.33, respectively. This strongly highlights the vital importance of interlayer charge transfer in the PCET step.Notably, these FPM‐based predictions fundamentally differ from those derived from the CHE method, where *c_d_‐c_p_* and c*_d_* are the dominant descriptors as described by *d*‐band center theory [45, 46], as shown in Figure S10. This discrepancy primarily arises from the fact that our FPM‐based analytical framework explicitly accounts for charge‐state variations under constant potential. The inclusion of this dynamic charge response introduces a crucial dimension for understanding electronic structure‐activity relationships, going beyond the limitations of conventional static descriptors.

In the following, we selected 14 features with Pearson correlation coefficient absolute values equal to or below 0.86 for predicting *∆G_N2_* and *∆G_NNH_* as presented in Figure 3b–g. Five supervised learning algorithms were employed for model training (also see Figures S2 and S3). The best‐performing regression model (LSBoost) with the highest performance for *∆G_N2_* (RMSE = 0.051 and R^2^ = 0.995) and *∆G_NNH_* (RMSE = 0.321 and R^2^ = 0.878), was selected for further analysis. To intuitively determine the impact of each feature, we employed the SHAP method. The distribution of SHAP values in Figure 3c shows a positive trend for *d_N_*_2_, confirming its direct correlation with Δ*G_N2_*, which aligns with the Pearson analysis. This relationship arises because enhanced electron transfer to N_2_ intensifies Coulombic repulsion with the negatively charged surface, thereby weakening adsorption. For the adsorption energy of N_2_ (see Figure 3d), the feature importance was ordered by mean absolute SHAP values. The top five features are ranked as follows: *d_N2_* > *Q_N2_* > *IE* > *c_d_* > *c_p_*. Crucially, the feature importance rank of the PCET step from ^*^N_2_ to ^*^NNH shifts to ∆P_*N2_ > *Q_g_* > *IE* > *c_d_* > *Q_N_*_2_ (see Figure 3f,g). This new ranking underscores the dominant role of PZCs in modulating reaction energetics. SHAP analyses were conducted for all five models and consistently identify PZCs as key across models (see Supporting Information, Note S2 and Figures S2–S3). In addition, the high conductivity of graphene facilitates significant interlayer charge redistribution, which profoundly alters the local electronic environment of the active site and is reflected in the importance of *Q_g_*.

It is worth noting that the limitations of the conventional CHE and the necessity of the FPM are starkly evident in ML. As shown in Figure S10, an interpretable ML model trained on CHE data incorrectly identifies the d‐band center descriptors (*c_d_*—*c_p_* and *c_d_*) as the most important for predicting ∆*G_N2_* and ∆*G_NNH_*, respectively. This flawed conclusion stems from a fundamental oversight in the CHE framework: it neglects the potential‐driven charge state variation between intermediate states (from *n*_1_ *e* to *n*_2_ *e*), which is governed by shifts in the PZCs. This distinction is crucial for SAMCs. Unlike conventional heterogeneous catalysts, where a diversity of surface adsorbates stabilizes the interface and minimizes PZC variation, SAMCs feature structurally simple and highly tunable active sites. Consequently, they are exquisitely sensitive to PZC shifts induced by the adsorption of specific intermediates. For SAMCs, voltage‐driven charge dynamics are not a minor effect but are pivotal, necessitating methods like FPM for a rigorous and comprehensive understanding of the NRR catalytic mechanism.

**FIGURE 4:**
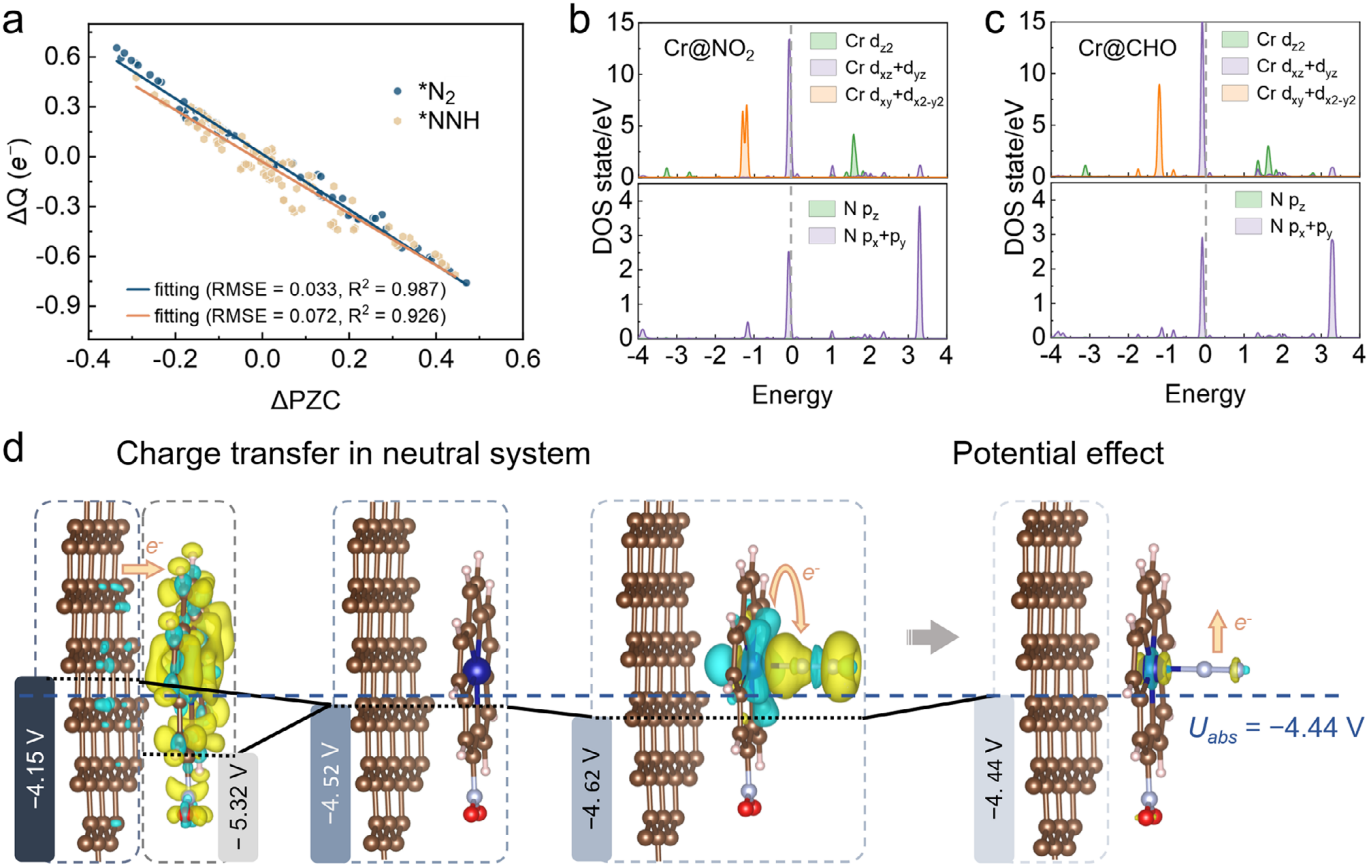
(a) Charge variation as a function of the PZC change before and after the reaction for *N_2_ and *NNH systems. PDOS of *N_2_ for (b) Cr@NO_2_‐carborin/graphene catalysts and (c) Cr@CHO‐carborin/graphene catalysts. The Fermi level is set to zero. (d) Schematic illustration of PZCs and applied potentials for the Cr@NO_2_‐carborin/graphene system in determining the charge transfer dynamics and its impact on the N_2_ activation. The first, third, and fourth panels indicate the charge transfer from graphene to NO_2_‐carborin, the charge transfer from the catalyst to the nitrogen molecule, and the charge variation induced by applied potential, respectively.

To deepen our understanding of the potential effect from an electronic structure perspective, we analyzed the relationship between charge and PZC, projected density of states (PDOS), and charge transfer processes as shown in Figure 4. Figure 4a reveals a strong linear correlation between the ∆Q and the ∆PZC for both ^*^N_2_ and ^*^NNH. The relationships are described by ∆Q_*N2_ = –1.670 ∆PZC_*N2_ + 0.016 (R^2^ = 0.987) and ∆Q_*NNH_ = –1.558 ∆PZC_*NNH_ – 0.031 (R^2^ = 0.926), respectively. The near‐identical slopes indicate that substituting different metal atoms and functional groups has only a minor influence on the system's intrinsic capacitance, which we estimate to be approximately 1.7‐1.8 e/V for the various states (Figure S11). Further insight comes from the electronic structure. The PDOS for ^*^N_2_ (Figure 4b,c) shows significant contributions from both the Cr‐*d* and N‐*p* orbitals near the Fermi level. The hybridization of these orbitals is crucial for N_2_ activation. This electronic interaction is also reflected in the machine learning model (Figure 3f), where the *c_d_* is identified as the third most important feature for the PCET step.

In Figure 4d, we constructed charge difference maps for neutral and charged systems to elucidate the collective behavior of electrons in the first two steps of the NRR. The mechanism unfolds in a sequence of charge redistribution events: First, the NO_2_‐carborin and graphene substrates form a composite structure via van der Waals interaction. Due to graphene's higher PZC (–4.15 V) compared to NO_2_‐carborin (–5.32 V), electrons transfer from graphene to the NO_2_‐carborin moiety. This is visually confirmed by charge depletion (cyan) over graphene and charge accumulation (yellow) over NO_2_‐carborin. Specifically, electrons migrate via interlayer coupling from the C‐*p_z_* orbitals of graphene to the π‐system of the NO_2_‐carborin aromatic rings and the Cr atom's *d_xz_*/*d_yz_* orbitals (with *z* defined as perpendicular to the catalyst plane). This redistribution stabilizes the system at an intermediate PZC of –4.52 V. Upon N_2_ adsorption, charge density around the Cr atom decreases, triggering a secondary electron migration from the Cr *d_xz_/d_yz_* orbitals into the *p_x_*, *p_y_*, and *p_z_* orbitals of the proximal N atom of N_2_. This facilitates the formation of *σ* and *π* bonds, while the distal N atom shows electron accumulation primarily in its *p_x_* and *p_y_* orbitals. Finally, under a constant applied potential (*U_abs_* = –4.44 V), significant electron accumulation occurs in the Cr *d_xz_/d_yz_* orbitals and the *p_x_/p_y_* orbitals of the distal N atom. This enrichment of electron density at the distal nitrogen is a crucial finding, as it directly promotes the hydrogenation kinetics and rationalizes the observed NNH bond angle of ∼112.55°—a key geometric descriptor for NRR catalytic activity.

Finally, we have supplemented some possible limitations of this work. While the high‐throughput screening strategy presented herein is broadly applicable to other catalyst families, the specific descriptor importance ranking derived from our explainable machine learning model is inherently tied to the coordination environment of the single carborane scaffold investigated. To exemplify this, we have performed a preliminary sensitivity check on two additional representative carbolong architectures (shown in Figure S5). The results indicate that the N_2_ adsorption energy increases from −0.98 eV for carborin (15C carbolong) to −0.38 eV for the 9C carbolong, confirming that these values are indeed sensitive to the atomic coordination environment. In addition, molecular catalysts differ from conventional bulk catalysts in their PZC behavior during the actual reaction process. For conventional bulk catalysts, their surfaces exhibit good electrical conductivity, and thus their PZC is primarily determined by the distribution and concentration of different reaction intermediates at the reaction interface. In contrast, molecular catalysts possess a limited number of active sites—for instance, single‐atom catalysts typically only allow the adsorption of a single intermediate at a given time. Consequently, changes in PZC exert a significant influence on the reaction activation energy, which supports our emphasis that PZC and PZC‐sensitive features act as the primary descriptors for NRR activity. Beyond the present computational screening space, our DFT+ML framework can be used to prioritize a small set of high‐confidence, synthesis‐feasible candidates, which can then be experimentally benchmarked under standardized electrochemical protocols. The resulting experimental labels can be incorporated into the training set to iteratively recalibrate and update the model, thereby improving predictive reliability beyond the original computational screening space [47].

## 4 Conclusion

This study elucidates the fundamental role of charge transfer dynamics and potential effects in electrochemical elementary steps by integrating the FPM with ML. We identify Cr@carborin/graphene‐based catalysts functionalized with nitro (NO_2_) or hydroxymethyl (CHO) groups as highly promising for the NRR. Our computational analysis reveals that variable charge states critically influence the PDS, a dependency captured by FPM but neglected by the conventional CHE approach. Furthermore, Pearson correlation and SHAP analysis establish the shift in the PZC during the first PCET step as a paramount descriptor. This is further supported by a robust linear relationship between PZC changes and system charge variations. Charge density visualizations clarify how PZC governs electron transfer, and they underscore that dynamic PZC optimization is essential for designing high‐performance catalysts. In summary, this work provides a deeper understanding of electrocatalytic mechanisms through a dual approach of data‐driven feature ranking and quantum‐chemical validation. It proposes systematic PZC optimization as a key design principle for efficient NRR catalysts, thereby bridging a critical theory‐experiment gap and accelerating the development of scalable electrochemical technologies.

## Conflicts of Interest

The authors declare no conflicts of interest.

## Supporting information




**Supporting File**: advs74631‐sup‐0001‐SuppMat.docx.

## Data Availability

The data that support the findings of this study are available in the supplementary material of this article.

## References

[1] J. W. Erisman , M. A. Sutton , J. Galloway , Z. Klimont , and W. Winiwarter , “How a Century of Ammonia Synthesis Changed the World,” Nature Geoscience 1 (2008): 636–639, 10.1038/ngeo325.

[2] T. Liu , X. Qu , Y. Zhang , et al., “Regulating the Charge Densities of S‐Block Calcium Single‐Atom Site Catalysts for Efficient N_2_ Activation and Reduction,” Chemical Engineering Journal 457 (2023): 141187, 10.1016/j.cej.2022.141187.

[3] C. Guo , D. Yang , M. Liang , X. Zhang , and H. Jiao , “Mechanistic Insights Into the Chemo‐Selective Dehydrogenative Silylation of Alkenes Catalyzed by Bis(imino)pyridine Cobalt Complex From DFT Computations,” Chemcatchem 12 (2020): 3890–3899, 10.1002/cctc.202000272.

[4] J. G. Chen , R. M. Crooks , L. C. Seefeldt , et al., “Beyond Fossil Fuel–Driven Nitrogen Transformations,” Science 360 (2018): aar6611, 10.1126/science.aar6611. PMC6088796 29798857

[5] F. Bird , A. Clarke , P. Davies , and E. Surkovic , Fuel and Energy Store (The Royal Society, 2020), 1–40.

[6] D. Yang , T. Chen , and Z. Wang , “Electrochemical Reduction of Aqueous Nitrogen (N_2_) at a Low Overpotential on (110)‐Oriented Mo Nanofilm,” Journal of Materials Chemistry A 5 (2017): 18967–18971, 10.1039/C7TA06139K.

[7] X. Cui , C. Tang , and Q. Zhang , “A Review of Electrocatalytic Reduction of Dinitrogen to Ammonia Under Ambient Conditions,” Advanced Energy Materials 8 (2018): 1800369, 10.1002/aenm.201800369.

[8] E. Skúlason , T. Bligaard , S. Gudmundsdóttir , et al., “A Theoretical Evaluation of Possible Transition Metal Electro‐Catalysts for N_2_ Reduction,” Physical Chemistry Chemical Physics 14 (2011): 1235–1245, 10.1039/C1CP22271F. 22146855

[9] M. M. Montemore and J. W. Medlin , “Scaling Relations Between Adsorption Energies for Computational Screening and Design of Catalysts,” Catalysis Science & Technology 4 (2014): 3748–3761, 10.1039/C4CY00335G.

[10] Y. Wu , C. He , and W. Zhang , “Capture‐Backdonation‐Recapture″ Mechanism for Promoting N 2 Reduction by Heteronuclear Metal‐Free Double‐Atom Catalysts,” Journal of the American Chemical Society 144 (2022): 9344–9353, 10.1021/jacs.2c01245. 35594427

[11] M. Luo , Y. Hua , K. Zhuo , et al., “Pentadentate Chelates by Formal [3+1] Cycloadditions of Metalla‐Azirines With Terminal Alkynes,” CCS Chemistry 3 (2020): 758–763, 10.31635/ccschem.020.202000223.

[12] J. Li , Z. Chu , Z. Lu , M. Luo , D. Chen , and H. Xia , “Reactivity Studies of a Hydroxy‐Substituted Irida‐Carbolong Complex,” Organometallics 41 (2022): 2589–2596, 10.1021/acs.organomet.2c00306.

[13] X. Lai , S. Chen , X. Gu , et al., “Phenanthroline‐Carbolong Interface Suppress Chemical Interactions With Active Layer Enabling Long‐Time Stable Organic Solar Cells,” Nature Communications 14 (2023): 3571, 10.1038/s41467-023-39223-9. PMC10272153 37322001

[14] G. Jia , “Facile Synthesis of Transition Metal Complexes With Five Coplanar Metal‐Carbon σ‐Bonds,” National Science Review 11 (2024): nwae078, 10.1093/nsr/nwae078. 38666093 PMC11044960

[15] Z. Deng , P. Wu , Y. Cai , et al., “Dioxygen Activation by Internally Aromatic Metallacycle: Crystallographic Structure and Mechanistic Investigations,” Iscience 23 (2020): 101379, 10.1016/j.isci.2020.101379. 32739835 PMC7399181

[16] B. Xu , D. Chen , K. Ruan , et al., “Metal‐Centred Planar [15]Annulenes,” Nature 641 (2025): 106–111, 10.1038/s41586-025-08841-2. 40307519

[17] G. Gao and L.‐W. Wang , “Protocol for Evaluating the Effect of Potential on Electrochemical Reactions via the Grand Canonical Fixed‐Potential Technique,” STAR Protocols 5 (2024): 103021, 10.1016/j.xpro.2024.103021. 38635396 PMC11043948

[18] Q. Zhang , Y. Zhang , S. Zhang , and G. Gao , “Potential Effects on the Catalytic Mechanisms of OER and ORR,” The Journal of Physical Chemistry C 127 (2023): 16346–16356, 10.1021/acs.jpcc.3c01984.

[19] G. Gao and L.‐W. Wang , “Substantial Potential Effects on Single‐Atom Catalysts for the Oxygen Evolution Reaction Simulated via a Fixed‐Potential Method,” Journal of Catalysis 391 (2020): 530–538, 10.1016/j.jcat.2020.08.032.

[20] Y. Wan , R. Li , J. Su , et al., “Single–Atom Supported Catalysts and Beyond,” Advanced Materials 37 (2025): 2504518, 10.1002/adma.202504518. 40468607

[21] O. Andreussi , I. Dabo , and N. Marzari , “Revised Self‐Consistent Continuum Solvation in Electronic‐Structure Calculations,” The Journal of Chemical Physics 136 (2012): 064102, 10.1063/1.3676407. 22360164

[22] Z. Wang , Y. Yang , D. L. Olmsted , M. Asta , and B. B. Laird , “Evaluation of the Constant Potential Method in Simulating Electric Double‐Layer Capacitors,” The Journal of Chemical Physics 141 (2014): 184102, 10.1063/1.4899176. 25399127

[23] W. Jia , Z. Cao , L. Wang , et al., “The Analysis of a Plane Wave Pseudopotential Density Functional Theory Code on a GPU Machine,” Computer Physics Communications 184 (2013): 9–18, 10.1016/j.cpc.2012.08.002.

[24] S. Nosé , “A Unified Formulation of the Constant Temperature Molecular Dynamics Methods,” Journal of Chemical Physics 81 (1984): 511–519, 10.1063/1.447334.

[25] W. G. Hoover , “Canonical Dynamics: Equilibrium Phase‐Space Distributions,” Physical Review A 31 (1985): 1695–1697, 10.1103/PhysRevA.31.1695. 9895674

[26] M. A. Spackman and P. G. Byrom , “A Novel Definition of a Molecule in a Crystal,” Chemical Physics Letters 267 (1997): 215–220, 10.1016/S0009-2614(97)00100-0.

[27] J. J. McKinnon , A. S. Mitchell , and M. A. Spackman , “Hirshfeld Surfaces: A New Tool for Visualising and Exploring Molecular Crystals,” Chemistry—A European Journal 4 (1998): 2136–2141, 10.1002/(SICI)1521-3765(19981102)4:11<2136.

[28] J. J. McKinnon , D. Jayatilaka , and M. A. Spackman , “Towards Quantitative Analysis of Intermolecular Interactions With Hirshfeld Surfaces,” Chemical Communications 37 (2007): 3814–3816, 10.1039/B704980C. 18217656

[29] M. A. Spackman and J. J. McKinnon , “Fingerprinting Intermolecular Interactions in Molecular Crystals,” CrystEngComm 4 (2002): 378–392, 10.1039/B203191B.

[30] J. J. McKinnon , M. A. Spackman , and A. S. Mitchell , “Novel Tools For Visualizing And Exploring Intermolecular Interactions In Molecular Crystals,” Acta Crystallographica Section B Structural Science 60 (2004): 627–668, 10.1107/S0108768104020300. 15534375

[31] J. K. Nørskov , J. Rossmeisl , A. Logadottir , et al., “Origin of the Overpotential for Oxygen Reduction at a Fuel‐Cell Cathode,” Journal of Physical Chemistry B 108 (2004): 17886–17892, 10.1021/jp047349j. 39682080

[32] Á. Valdés , Z.‐W. Qu , G.‐J. Kroes , J. Rossmeisl , and J. K. Nørskov , “Oxidation and Photo‐Oxidation of Water on TiO2 Surface,” Journal of Physical Chemistry C 112 (2008): 9872–9879, 10.1021/jp711929d.

[33] R. Johnson , “Computational Chemistry Comparison and Benchmark Database,” NIST Standard Reference Database 101 (2002), 10.18434/T47C7Z.

[34] G. Gao and L.‐W. Wang , “The Concerted Proton‐Electron Transfer Mechanism of Proton Migration in the Electrochemical Interface,” Iscience 26 (2023): 108318, 10.1016/j.isci.2023.108318. 38026153 PMC10661362

[35] Y. Xue , L.‐W. Wang , and G. Gao , “Presolvation Dynamics Preceding the Hydrated Proton Transfer in the Electrical Double Layer,” The Journal of Physical Chemistry Letters 16 (2025): 4572–4578, 10.1021/acs.jpclett.5c00721. 40309954

[36] L. Breiman , “Random Forests,” Machine learning 45 (2001): 5–32, 10.1023/A:1010933404324.

[37] Y. Freund and R. E. Schapire , “A Decision‐Theoretic Generalization of on‐Line Learning and an Application to Boosting,” Journal of Computer and System Sciences 55 (1997): 119–139, 10.1006/jcss.1997.1504.

[38] S. M. Lundberg and S.‐I. Lee , “A Unified Approach to Interpreting Model Predictions,” Proceedings of the 31st International Conference on Neural Information Processing Systems (2017), 4768–4777, https://dl.acm.org/doi/10.5555/3295222.3295230.

[39] K. Aas , M. Jullum , and A. Løland , “Explaining Individual Predictions When Features Are Dependent: More Accurate Approximations to Shapley Values,” Artificial Intelligence 298 (2021): 103502, 10.1016/j.artint.2021.103502.

[40] S. M. Lundberg , G. Erion , H. Chen , et al., “From Local Explanations to Global Understanding With Explainable AI for Trees,” Nature Machine Intelligence 2 (2020): 56–67, 10.1038/s42256-019-0138-9. PMC7326367 32607472

[41] J. Yu , X. Yong , and S. Lu , “p‐d Orbital Hybridization Engineered Single‐Atom Catalyst for Electrocatalytic Ammonia Synthesis,” Energy & Environmental Materials 7 (2024): 12587, 10.1002/eem2.12587.

[42] H. Yin , S.‐L. Li , L.‐Y. Gan , and P. Wang , “Pt‐Embedded in Monolayer G‐C_3_N_4_ as a Promising Single‐Atom Electrocatalyst for Ammonia Synthesis,” Journal of Materials Chemistry A 7 (2019): 11908–11914, 10.1039/C9TA01624D.

[43] C. He , D. Chen , and W. X. Zhang , “Machine Learning‐Driven Shortening the Screening Process towards High‐Performance Nitrogen Reduction Reaction Electrocatalysts With Four‐Step Screening Strategy,” Journal of Colloid and Interface Science 676 (2024): 22–32, 10.1016/j.jcis.2024.07.109. 39018807

[44] A. R. Singh , B. A. Rohr , J. A. Schwalbe , et al., “Electrochemical Ammonia Synthesis—The Selectivity Challenge,” ACS Catalysis 7 (2017): 706–709, 10.1021/acscatal.6b03035.

[45] J. K. Nørskov , “Theory Nof Chemisorption and Heterogeneous Catalysis,” Phys BC 127 (1984): 193–202, 10.1016/S0378-4363(84)80030-3.

[46] A. Ruban , B. Hammer , P. Stoltze , H. L. Skriver , and J. K. Nørskov , “Surface electronic structure and reactivity of transition and noble metals1Communication presented at the First Francqui Colloquium, Brussels, 19–20 February 1996.1,” Journal of Molecular Catalysis A: Chemical 115 (1997): 421–429, 10.1016/S1381-1169(96)00348-2.

[47] K. Lee , M. Han , S. J. Lee , et al., “Standardization and Machine Learning Prediction of Tafel Slope of Pt‐Based Nanocatalysts for High‐Performance HER Catalyst Development,” Small Methods 10 (2026): 01909, 10.1002/smtd.202501909. 41467523

