# Supplementary material for: Integrating Machine Learning With Constant‐Potential Simulation to Unravel Charge‐Transfer Mechanisms in Electrochemical Nitrogen Fixation
Source: Adv Sci (Weinh). 2026 Mar 13:e24356. Online ahead of print. doi: 10.1002/advs.202524356 (PMC13325948; doi:10.1002/advs.202524356)
Supplement: Supplementary file 1 — Supporting File: advs74631‐sup‐0001‐SuppMat.docx. [file ADVS-9999-e24356-s001.docx]

**Integrating Machine Learning with Constant-Potential Simulation to Unravel Charge-Transfer Mechanisms in Electrochemical Nitrogen Fixation**

Yufei Xue^1^, DushuoFeng^2^, Yuefei Zhang^1^, Yang Zhang^1^, Yalong Jiao^3*^, Aijun Du^4*^, Guoping Gao^1*^

1 MOE Key Laboratory for Non-equilibrium Synthesis and Modulation of Condensed Matter, Shaanxi Province Key Laboratory of Advanced Functional Materials and Mesoscopic Physics, School of Physics, Xi’an Jiaotong University, Xi’an, Shaanxi 710049, China; E-mail: [guopinggao@xjtu.edu.cn](mailto:guopinggao@xjtu.edu.cn)

2 Department of Sport and Exercise Science, College of Education, Zhejiang University, Hangzhou 310058, China

3 College of Physics, Hebei Key Laboratory of Photophysics Research and Application, Hebei Normal University, Shijiazhuang 050024, China. E-mail: yalong.jiao@hebtu.edu.cn

4 School of Chemistry and Physics and Centre for Material Science, Faculty of Science, Queensland University of Technology, Gardens Point Campus, Brisbane, Queensland 4001, Australia; Email: aijun.du@qut.edu.au

**Note S1. Details of the DFT Calculations**

All the DFT calculations of structural relaxation, free energies of elementary steps and *ab initio* molecular dynamics (AIMD) were conducted using the PWmat package incorporating the grand canonical fixed-potential methond (FPM) ^1,2^. These calculations employed the generalized gradient approximation (GGA), specifically in the form of the Perdew-Burke-Ernzerhof (PBE) functional, along with the SG15 norm-conserving pseudopotential ^3–5^. For structural relaxation, the energy convergence threshold and the tolerance for the residual force acting on each atom were established at 10^-5^ eV and 0.02 eV Å^-1^, respectively. The electronic wave functions were represented through an expansion in plane waves, employing a cutoff energy of 40 Ry, and the Brillouin zone was sampled with the Monkhorst-Pack mesh with a k-point of 2 × 2 × 1. The lattice constant of the unit cell along the Z direction is set to 20 Å, resulting in a vacuum thickness of approximately 16 Å when the catalyst is present. As validated in our previous work, this vacuum thickness is sufficient to shield spurious periodic interactions in DFT calculations^6^. For free energy calculations, Grimme’s DFT-D3 dispersion correction scheme was incorporated to account for van der Waals interactions. In the kinetic stability testing, we performed AIMD simulations with a 1 fs timestep and a Γ-centered 1 × 1 × 1 k-point was used for Brillouin zone sampling, maintaining the temperature at 300 K via Nosé–Hoover thermostats in the canonical ensemble ^7,8^.

To ensure we identified the most stable structure and explicitly confirm that the optimized structure corresponds to the global minimum energy configuration, we systematically tested two typical initial placements for the N_2_ molecule on the active site. Specifically, regarding the orientation of N_2_, we found that the initial configuration with N_2_ parallel to the carborin plane is unstable during structural optimization, it spontaneously relaxes to a perpendicular orientation relative to the carborin moiety. Take Cr@Carborin as an example, the calculated adsorption energy for the perpendicular configuration is significantly lower than that of the parallel state about 0.69 eV, confirming the perpendicular orientation as the thermodynamic ground state.

Additionly, our high-throughput density functional theory (DFT) screening was mainly carried out under standard conditions (pH = 0) using implicit solvation models to ensure data consistency and stability across the sample space. Additionally, the influence of pH was implicitly handled through the computational hydrogen electrode (CHE) model, and a specific correction term (ΔG_pH_ = −*k_BT_* × ln (10) × pH) was applied to reaction steps involving proton transfer. Figure S1 presents Gibbs free energy diagrams for the two optimal NRR candidates at pH 0, 7, and 14. These results demonstrate that while increasing pH raises the free energy of proton-involved steps, the potential-determining steps and the significant energetic preference for NRR over HER remain robust. Moreover, the identified catalysts can still maintain excellent NRR selectivity even in acidic and neutral media.


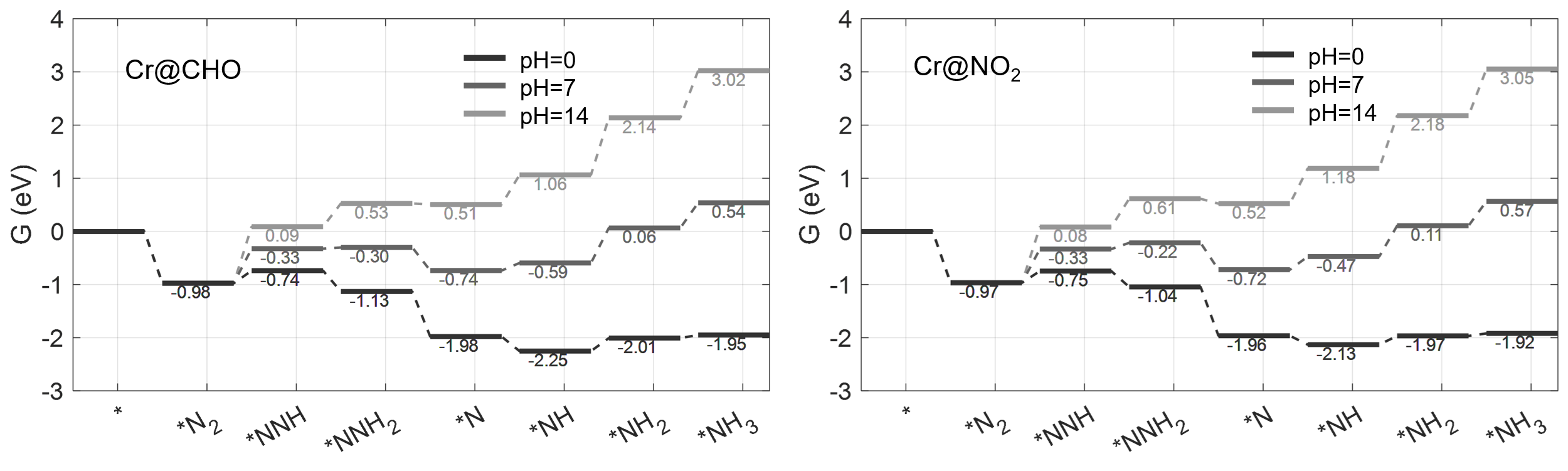


**Fig. S1** The Gibbs free energy diagrams of NRR pathways for two best catalysts (Cr@NO2-carborin and Cr@CHO-carborin) obtained by FPM at *U_abs_* = –4.44 V

**Note S2. Testing of the Machine Learning Models**

The five supervised learning regression models used in our work are Gaussian Kernel Regression ^9–11^, Linear Regression ^12,13^, Support Vector Machine regression ^14–16^, Least Squares Boosting Regression ^17,18^, and Gaussian Process Regression ^19,20^. Efficient and reliable ML models, capable of nonlinear processing, are essential for uncovering potential effects in catalysis. Therefore, we selected 80% of DFT data for training and the remaining 20% for predicting ∆G_N2_ and ∆G_NNH_. The Root Mean Square Error (RMSE) and the Coefficient of Determination (R² score) are shown in Figs. S2 and S3. Among these, Least Squares Boosting Regression models (RMSE=0.051 and R^2^ =0.995 for *∆G_N2_* prediction, and RMSE=0.321 and R^2^ =0.878 for *∆G_NNH_* prediction) are particularly favored due to their robustness and interpretability, making them widely adopted in this field. To further assess model robustness beyond a single train-test split, we additionally performed 5-fold cross-validation for the best-performing regression model (LSBoost). The fold-wise and averaged metrics are summarized in Table S1. Overall, the cross-validation results confirm stable predictive performance for both ∆G_N2_ and ∆G_NNH_, supporting the reliability of the trained models.


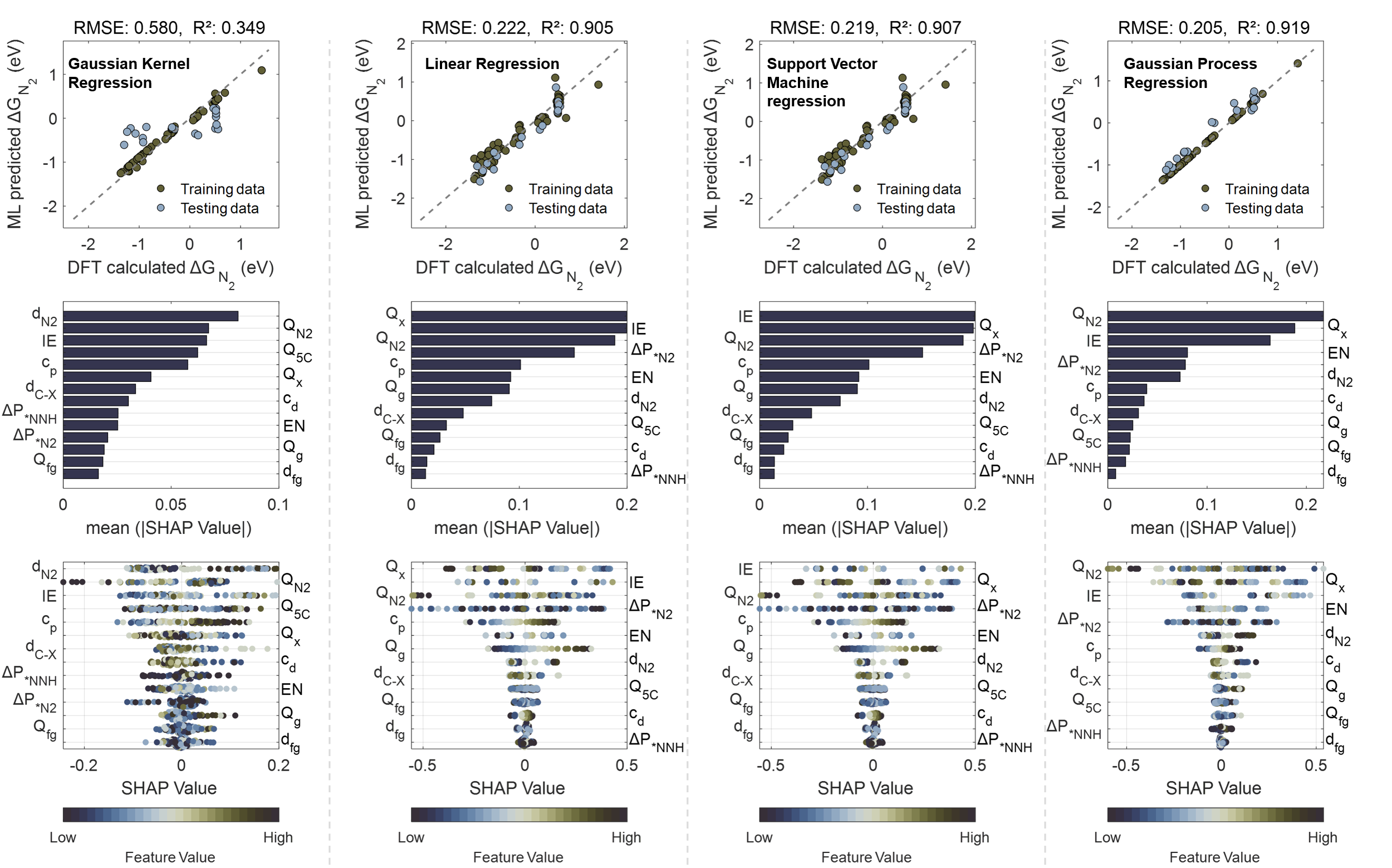


**Fig. S2** Prediction performance, mean SHAP values, and distribution of SHAP values for predicting ∆G_N2_ obtained by the supervised learning regression models: Gaussian Kernel Regression, Linear Regressio, Support Vector Machine regressio, and Gaussian Process Regression, respectively.


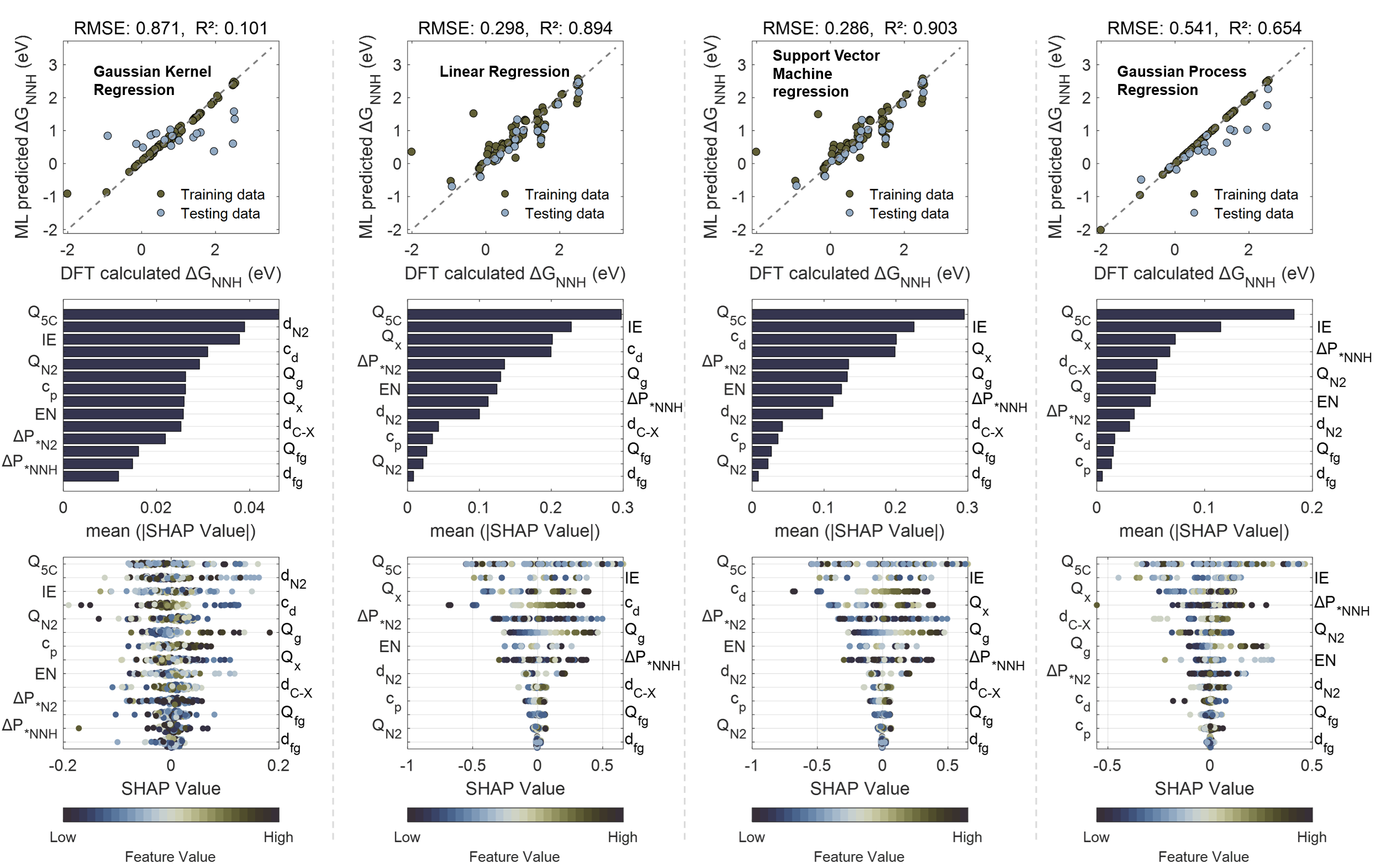


**Fig. S3** Prediction performance, mean SHAP values, and distribution of SHAP values for predicting ∆G_NNH_ obtained by the five supervised learning regression models.

**Table. S1** 5-fold cross-validation of five models for predicting ∆G_N2_ and ∆G_NNH_.

| **5-fold cross-validation** | | **Fold 1** | **Fold 2** | **Fold 3** | **Fold 4** | **Fold 5** | **Average** |
| --- | --- | --- | --- | --- | --- | --- | --- |
| ∆G_N2_ | RMSE | 0.0500 | 0.0502 | 0.0514 | 0.0521 | 0.0884 | 0.0584 |
|  | R^2^ | 0.9942 | 0.9943 | 0.9941 | 0.9935 | 0.9863 | 0.9924 |
| ∆G_NNH_ | RMSE | 0.1974 | 0.1778 | 0.1850 | 0.1959 | 0.2434 | 0.1998 |
|  | R^2^ | 0.9213 | 0.9280 | 0.9202 | 0.9044 | 0.9266 | 0.9200 |

**Note S3. Faradaic efficiency of NRR**

 To effectively evaluate the selectivity of NRR over HER, the following assumptions are made: first, HER is considered the only competing reaction; second, neither the transfer of protons nor electrons serves as the rate-determining step in either HER or NRR. Based on these assumptions, the relative selectivity of NRR with respect to HER can be reasonably approximated by employing the Boltzmann distribution. Consequently, under such specified conditions, the Faradaic efficiency (FE) of NRR can be determined by

$$FE=\frac{1}{1+e^{-\frac{\Delta G}{k_{B}T}}}\times100\%$$

where ∆G is the Gibbs free energy difference between the adsorption energies of *H and *N_2_, *k_B_* and *T* are the Boltzmann constant and room temperature values^21,22^. The constant-potential effect has been considered for HER and NRR calculations. This consideration ensures the consistency of the main content of the article.


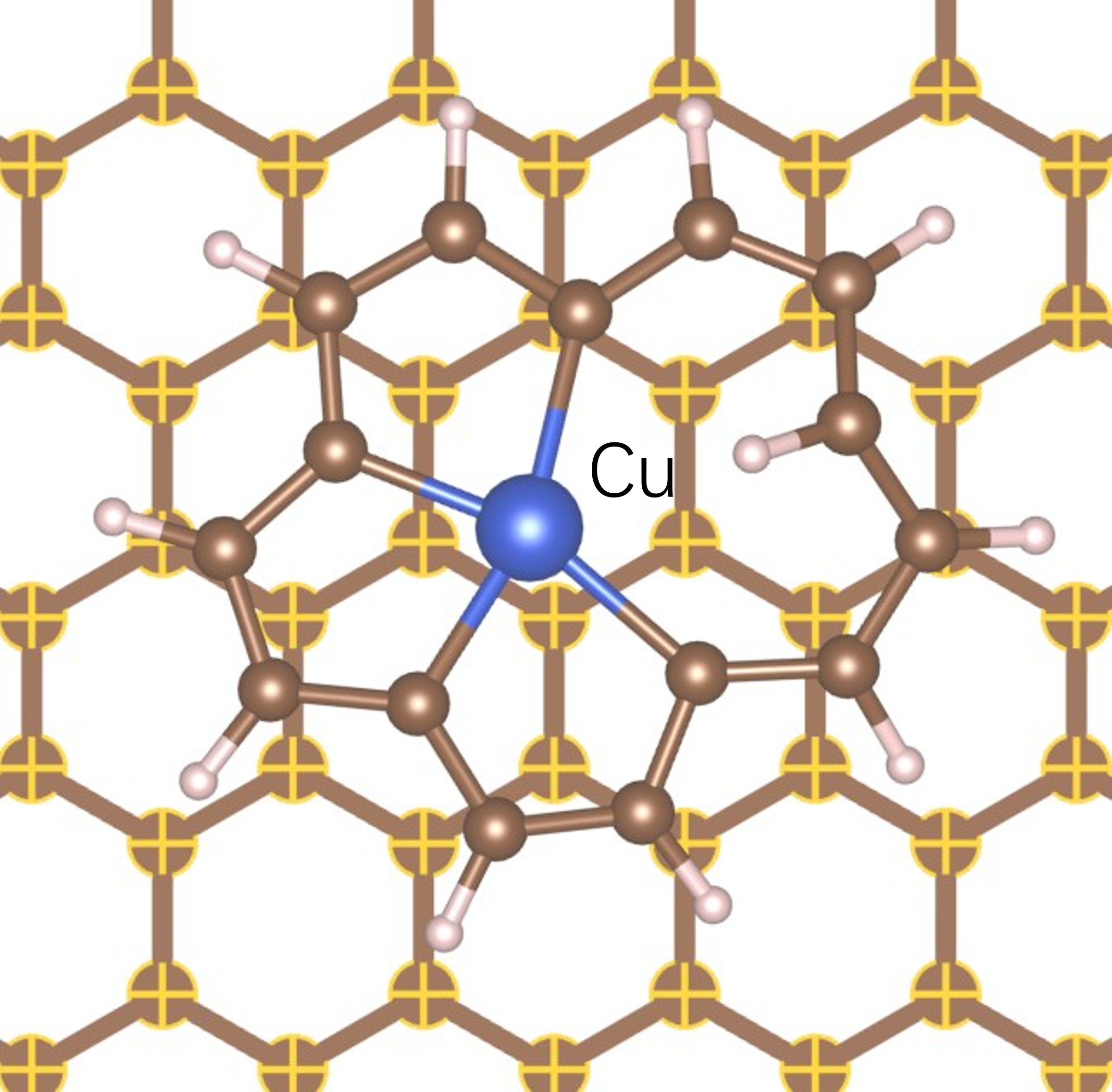


**Fig. S4** Optimized structure of Cu@carborin. The atoms highlighted in yellow represent the graphene substrate.


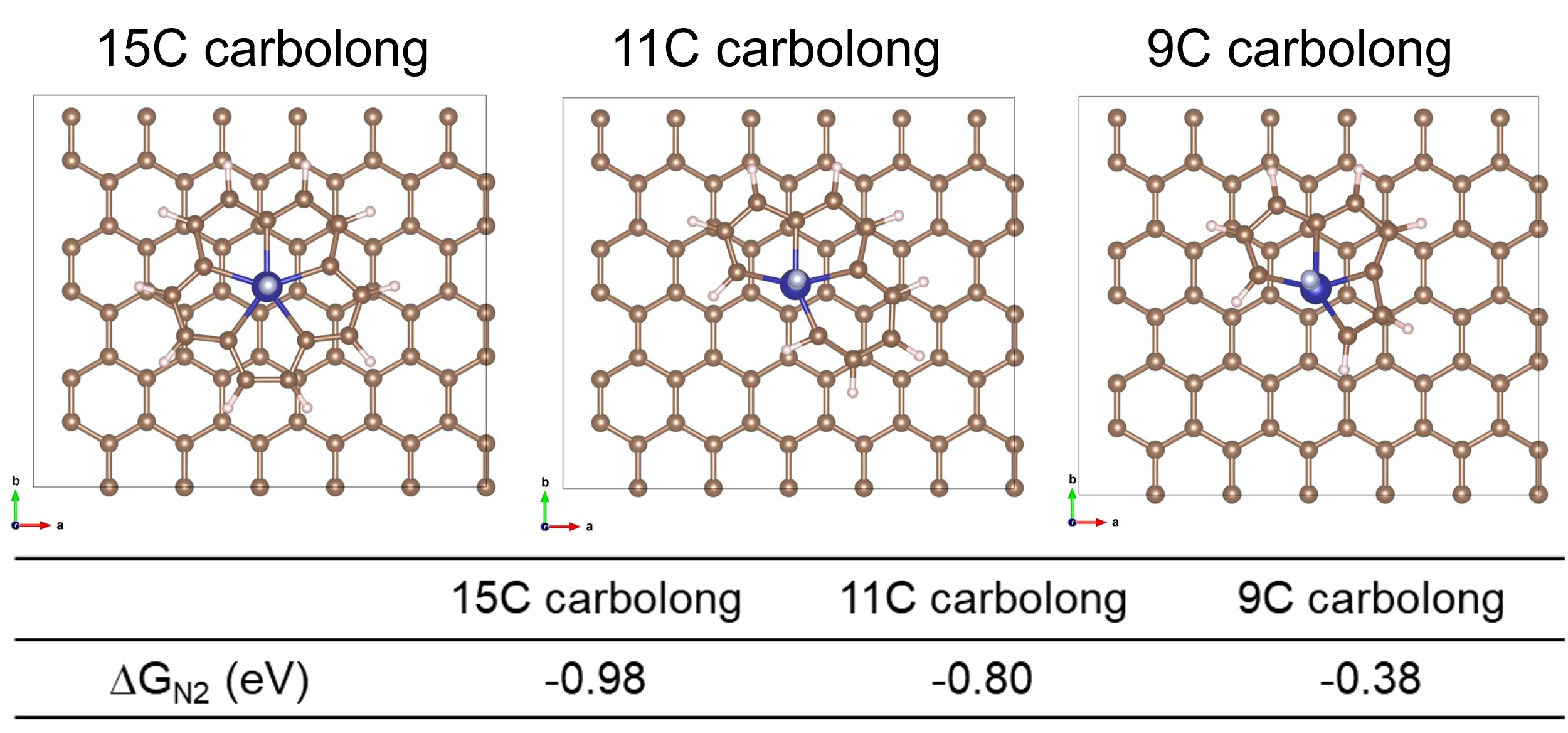


**Fig. S5** The top views and N_2_ adsorption energies of three carbolongs.


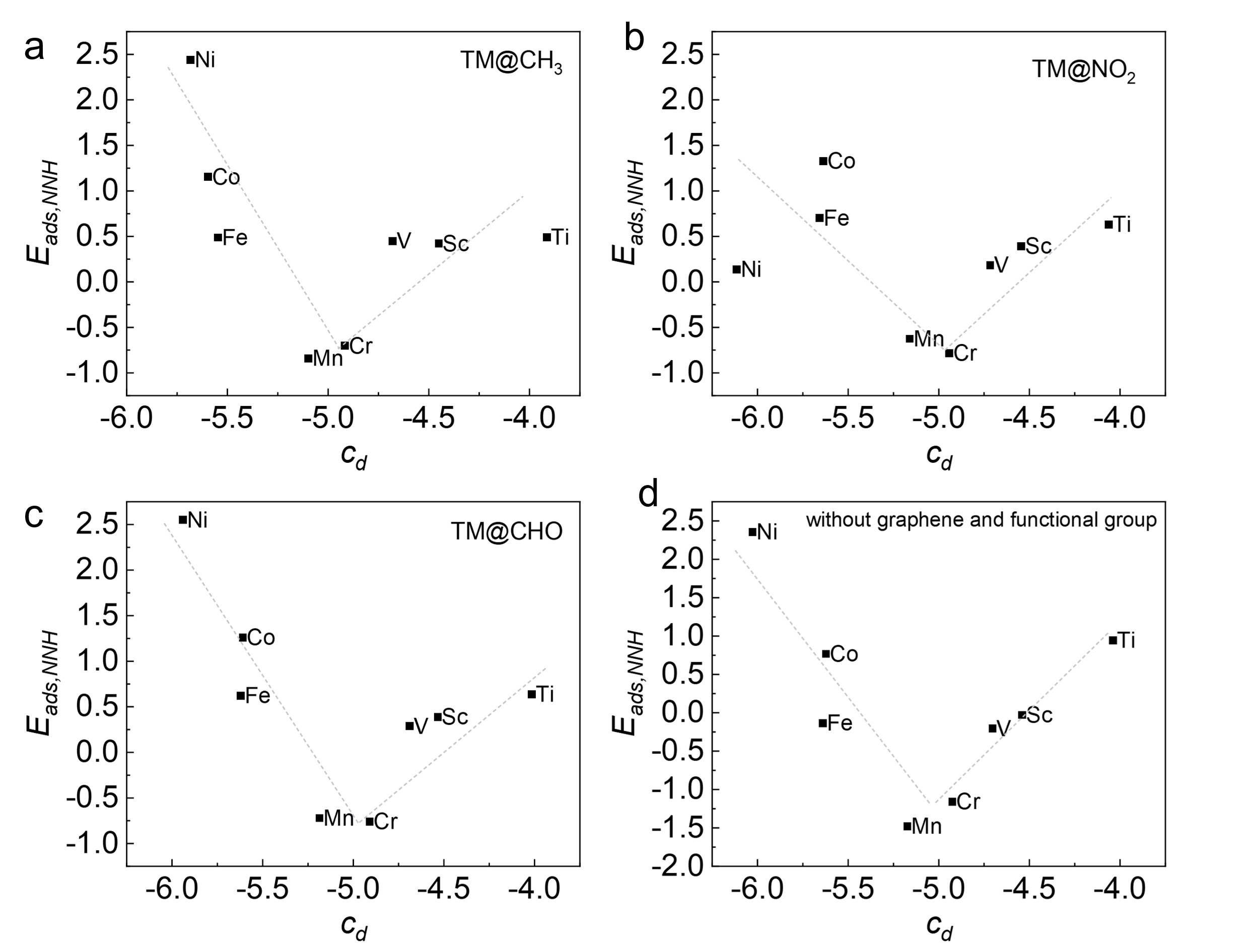


**Fig. S6** *NNH adsorption energy versus metal *d*-band center in TM@carborin-graphene (TM = Sc–Ni) with (a) CH_3_, (b) NO_2_ and (c) CHO groups, and (d) catalysts without functional groups and graphene support.


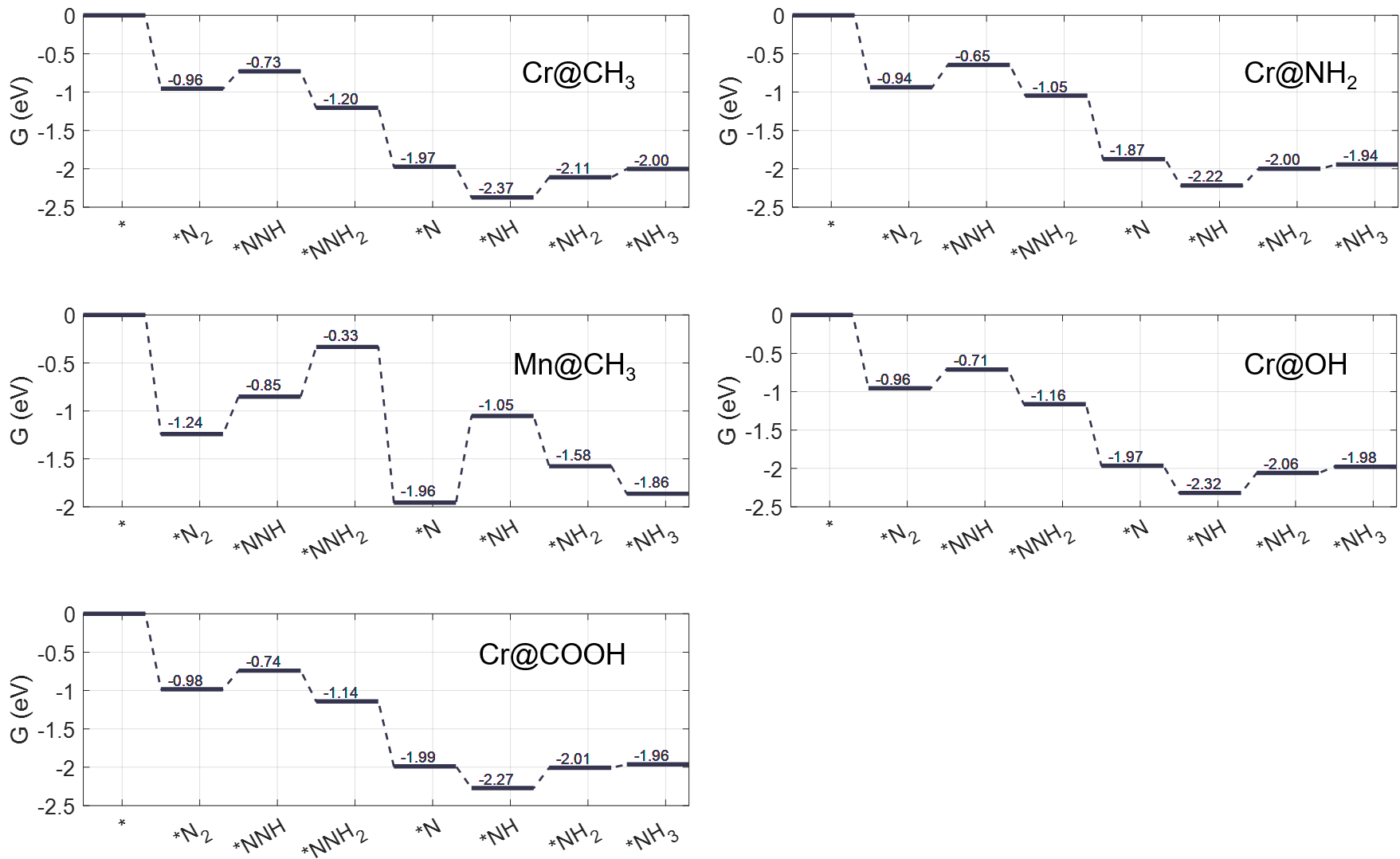


**Fig. S7** The free energy diagrams obtained by FPM at *U* = 0 V (*vs.* SHE) for the nitrogen reduction reaction on the graphene supported Cr@CH_3_-carborin, Mn@CH_3_-carborin, Cr@COOH-carborin, Cr@NH_2_-carborin, and Cr@OH-carborin, respectively.


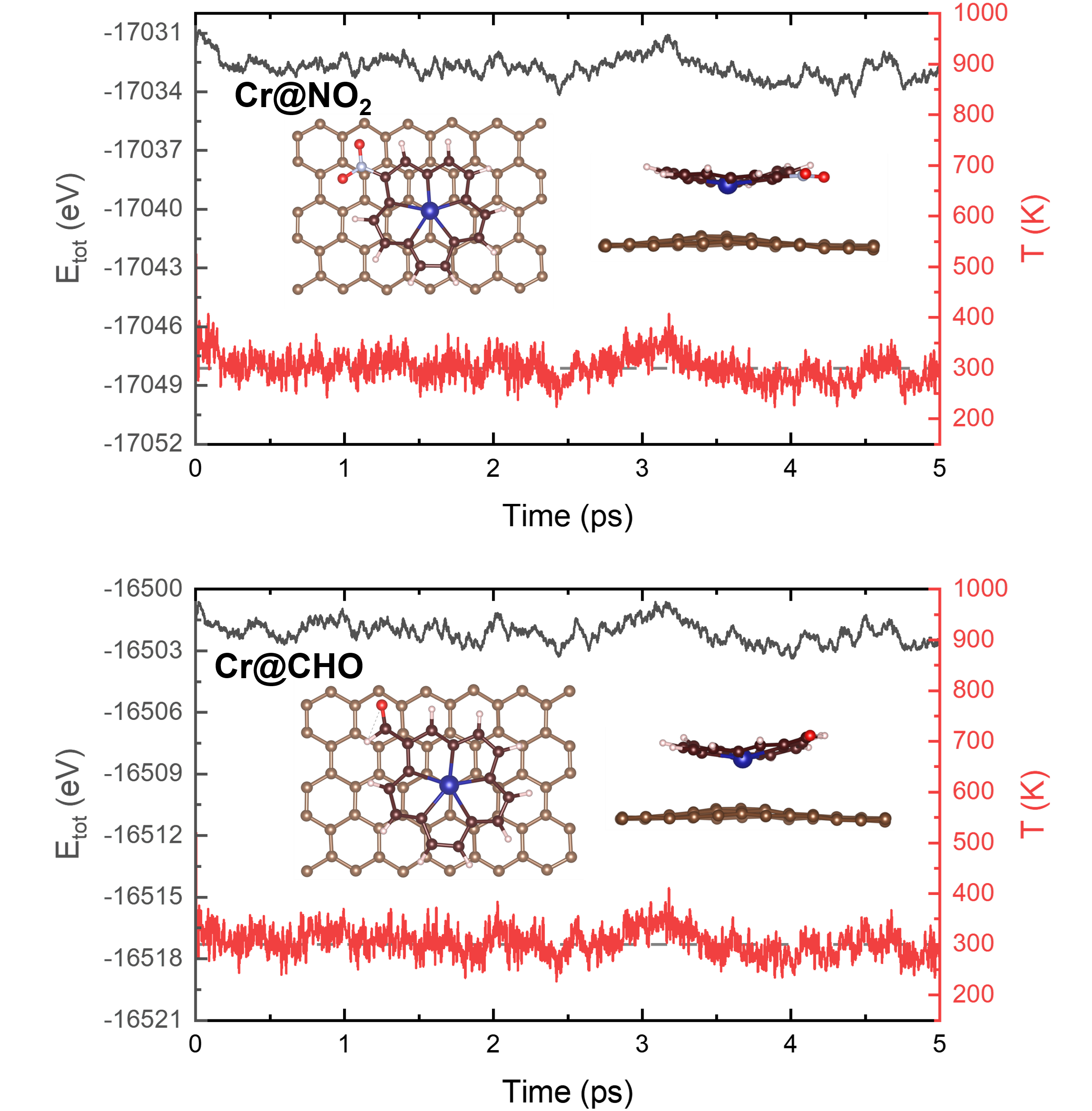


**Fig. S8** Total energy as a function of simulation time for Cr@NO_2_-carborin/graphene and Cr@CHO-carborin/graphene, performed at 500 K over 10 ps using *ab initio* molecular dynamics.


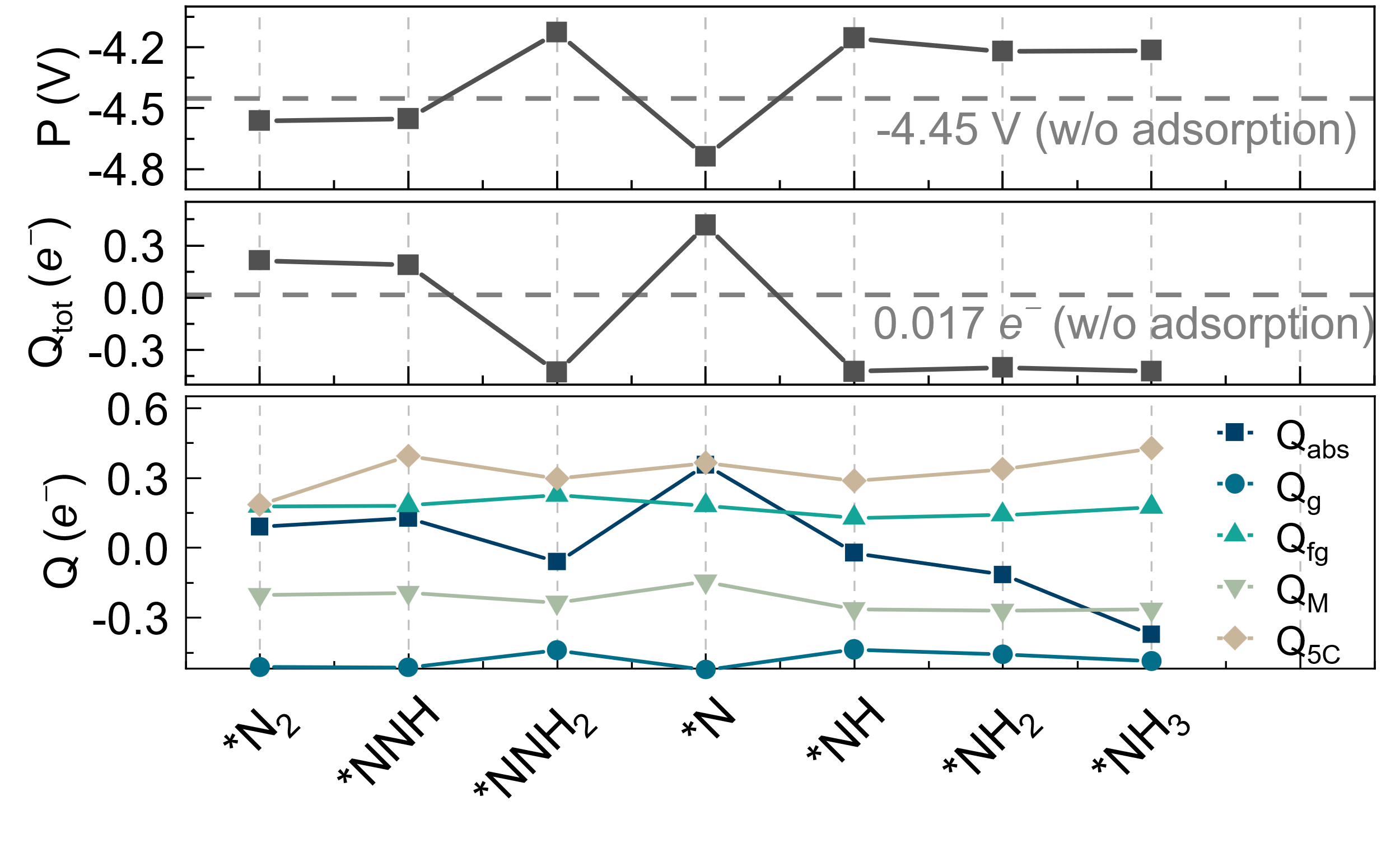


**Fig. S9** Potential of zero charge *P* and charge states *Q* of intermediates for the Gr@COH-carborin/graphene.


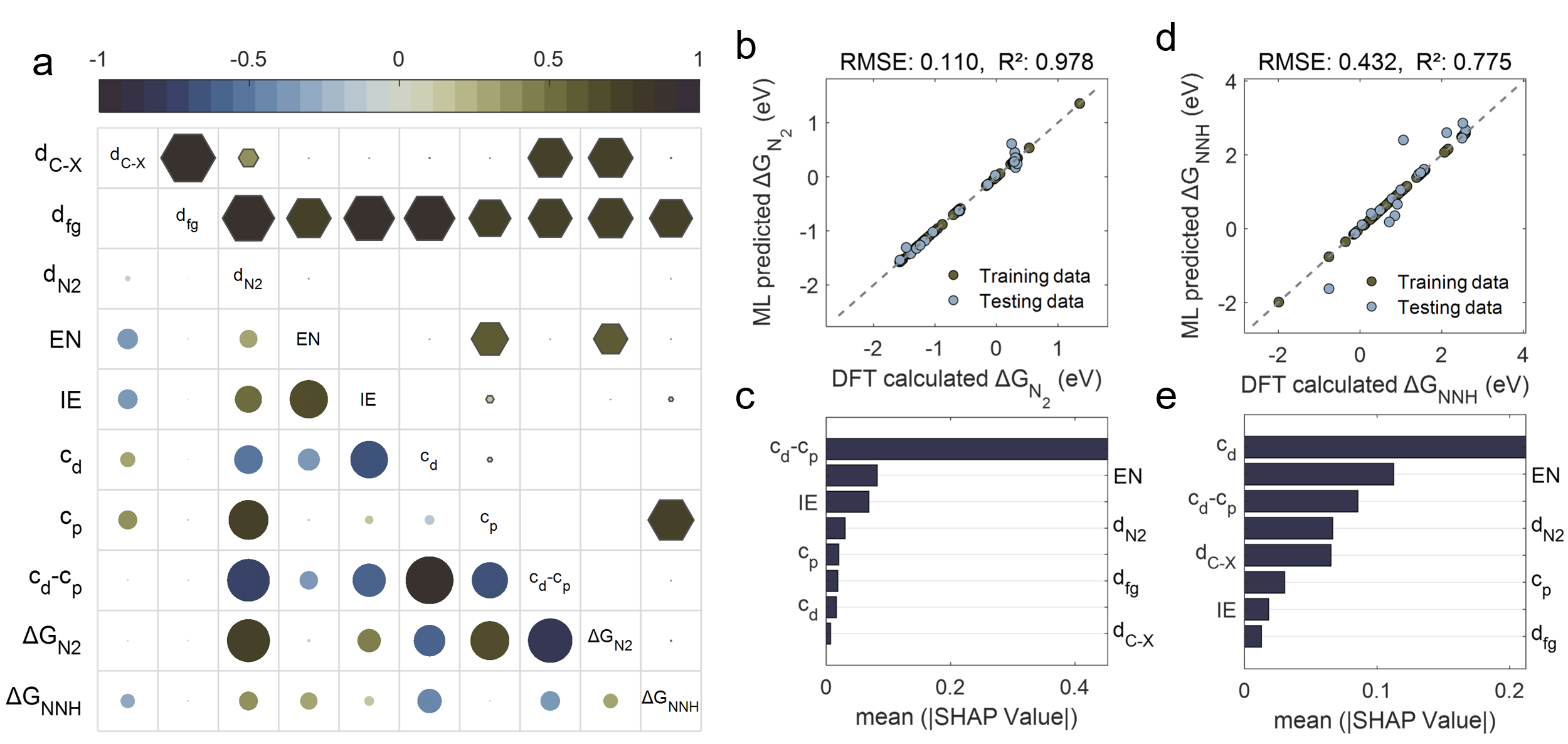


**Fig. S10** (a) Pearson correlation coefficients obtained by computational hydrogen electrode (CHE) approach. (b) Prediction performance for N_2_ adsorption energy obtained by Least Squares Boosting Regression model and CHE. (c) The mean SHAP values for each feature in predicting N_2_ adsorption energy. (d)-(e) Prediction performance and SHAP analysis for free energy difference of the PECT step from *N_2_ to *NNH.


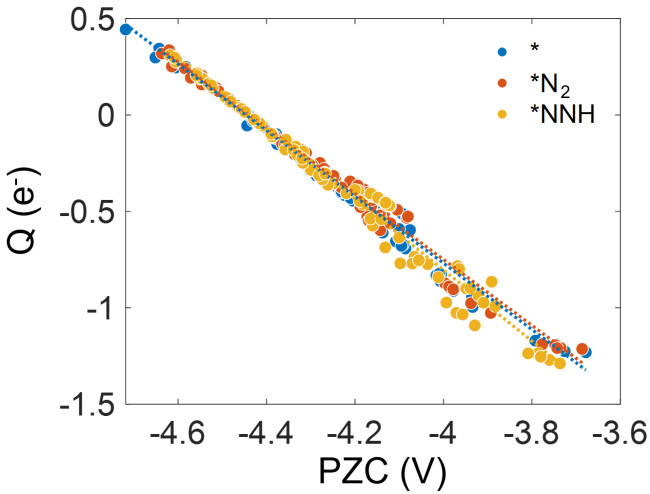


**Fig. S11** Charge variation compared with charge-neutral systems as a function of the pential of zero charge for *, *N_2_, and *NNH systems obtained by FPM under *U_abs_* = −4.44 V.


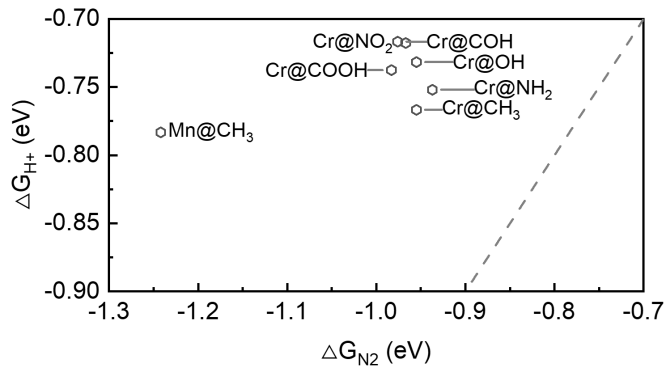


**Fig. S12** Adsorption energies of N_2_ and H^+^ obtained by FPM under *U_abs_* = −4.44 V.

References

(1) Wang, Z.; Yang, Y.; Olmsted, D. L.; Asta, M.; Laird, B. B. Evaluation of the Constant Potential Method in Simulating Electric Double-Layer Capacitors. *J. Chem. Phys.* **2014**, *141* (18), 184102. https://doi.org/10.1063/1.4899176.

(2) Jia, W.; Cao, Z.; Wang, L.; Fu, J.; Chi, X.; Gao, W.; Wang, L.-W. The Analysis of a Plane Wave Pseudopotential Density Functional Theory Code on a GPU Machine. *Comput. Phys. Commun.* **2013**, *184* (1), 9–18. https://doi.org/10.1016/j.cpc.2012.08.002.

(3) Perdew, J. P.; Burke, K.; Ernzerhof, M. Generalized Gradient Approximation Made Simple. *Phys. Rev. Lett.* **1996**, *77* (18), 3865–3868. https://doi.org/10.1103/PhysRevLett.77.3865.

(4) Perdew, J. P.; Ernzerhof, M.; Burke, K. Rationale for Mixing Exact Exchange with Density Functional Approximations. *J. Chem. Phys.* **1996**, *105* (22), 9982–9985. https://doi.org/10.1063/1.472933.

(5) Grimme, S. Semiempirical GGA-type density functional constructed with a long-range dispersion correction. *J. Comput. Chem.* **2006**, *27* (15), 1787–1799. https://doi.org/10.1002/jcc.20495.

(6) Gao, G.; Wang, L.-W. The Concerted Proton-Electron Transfer Mechanism of Proton Migration in the Electrochemical Interface. *iScience* **2023**, *26* (11), 108318. https://doi.org/10.1016/j.isci.2023.108318.

(7) Nosé, S. A Unified Formulation of the Constant Temperature Molecular Dynamics Methods. *J. Chem. Phys.* **1984**, *81* (1), 511–519. https://doi.org/10.1063/1.447334.

(8) Hoover, W. G. Canonical Dynamics: Equilibrium Phase-Space Distributions. *Phys. Rev. A* **1985**, *31* (3), 1695–1697. https://doi.org/10.1103/PhysRevA.31.1695.

(9) Rahimi, A.; Recht, B. Random Features for Large-Scale Kernel Machines. In *Proceedings of the 21st International Conference on Neural Information Processing Systems*; NIPS’07; Curran Associates Inc.: Red Hook, NY, USA, 2007; pp 1177–1184.

(10) Le, Q.; Sarlós, T.; Smola, A. Fastfood: Approximating Kernel Expansions in Loglinear Time. In *Proceedings of the 30th International Conference on International Conference on Machine Learning - Volume 28*; ICML’13; JMLR.org: Atlanta, GA, USA, 2013; p III-244-III–252.

(11) Huang, P.-S.; Avron, H.; Sainath, T. N.; Sindhwani, V.; Ramabhadran, B. Kernel Methods Match Deep Neural Networks on TIMIT. In *2014 IEEE International Conference on Acoustics, Speech and Signal Processing (ICASSP)*; 2014; pp 205–209. https://doi.org/10.1109/ICASSP.2014.6853587.

(12) Ho, C.-H.; Lin, C.-J. Large-Scale Linear Support Vector Regression. *J Mach Learn Res* **2012**, *13* (1), 3323–3348. https://doi.org/https://dl.acm.org/doi/abs/10.5555/2503308.2503348.

(13) Hsieh, C.-J.; Chang, K.-W.; Lin, C.-J.; Keerthi, S. S.; Sundararajan, S. A Dual Coordinate Descent Method for Large-Scale Linear SVM. In *Proceedings of the 25th international conference on Machine learning*; ICML ’08; Association for Computing Machinery: New York, NY, USA, 2008; pp 408–415. https://doi.org/10.1145/1390156.1390208.

(14) Seko, A. Machine Learning with Systematic Density-Functional Theory Calculations: Application to Melting Temperatures of Single- and Binary-Component Solids. *Phys. Rev. B* **2014**, *89* (5). https://doi.org/10.1103/PhysRevB.89.054303.

(15) Fan, R.-E.; Chen, P.-H.; Lin, C.-J. Working Set Selection Using Second Order Information for Training Support Vector Machines. *J Mach Learn Res* **2005**, *6*, 1889–1918. https://doi.org/https://dl.acm.org/doi/10.5555/1046920.1194907.

(16) Kecman, V.; Huang, T.-M.; Vogt, M. Iterative Single Data Algorithm for Training Kernel Machines from Huge Data Sets: Theory and Performance. In *Support Vector Machines: Theory and Applications*; Wang, L., Ed.; Springer: Berlin, Heidelberg, 2005; pp 255–274. https://doi.org/10.1007/10984697_12.

(17) Breiman, L. Random Forests. *Mach. Learn.* **2001**, *45* (1), 5–32. https://doi.org/10.1023/A:1010933404324.

(18) Freund, Y.; Schapire, R. E. A Decision-Theoretic Generalization of On-Line Learning and an Application to Boosting. *J. Comput. Syst. Sci.* **1997**, *55* (1), 119–139. https://doi.org/10.1006/jcss.1997.1504.

(19) Quiñonero-Candela, J.; Rasmussen, C. E. A Unifying View of Sparse Approximate Gaussian Process Regression. *J Mach Learn Res* **2005**, *6*, 1939–1959. https://doi.org/https://jmlr.org/papers/volume6/quinonero-candela05a/quinonero-candela05a.pdf.

(20) Rasmussen, C. E.; Williams, C. K. I. Gaussian Processes for Machine Learning. **2005**, 1. https://doi.org/https://doi.org/10.7551/mitpress/3206.003.0004.

(21) Zhao, W.; Zhang, L.; Luo, Q.; Hu, Z.; Zhang, W.; Smith, S.; Yang, J. Single Mo1(Cr1) Atom on Nitrogen-Doped Graphene Enables Highly Selective Electroreduction of Nitrogen into Ammonia. *ACS Catal.* **2019**, *9* (4), 3419–3425. https://doi.org/10.1021/acscatal.8b05061.

(22) Liu, X.; Jiao, Y.; Zheng, Y.; Jaroniec, M.; Qiao, S.-Z. Building Up a Picture of the Electrocatalytic Nitrogen Reduction Activity of Transition Metal Single-Atom Catalysts. *J. Am. Chem. Soc.* **2019**, *141* (24), 9664–9672. https://doi.org/10.1021/jacs.9b03811.
